# Transformation of engineered nanomaterials through the prism of silver sulfidation†

**DOI:** 10.1039/c8na00103k

**Published:** 2018-08-17

**Authors:** Fan Zhang, Andrew J. Allen, Aaron C. Johnston-Peck, Jingyu Liu, John M. Pettibone

**Affiliations:** Materials Measurement Science Division, National Institute of Standards and Technology Gaithersburg MD 20899 USA fan.zhang@nist.gov john.pettibone@nist.gov

## Abstract

Understanding the structure transformation of engineered nanomaterials (ENMs) is a grand measurement challenge, which impacts many aspects of ENMs applications, such as their efficacy, safety, and environmental consequence. To address the significant knowledge gap regarding the fundamental kinetic rate and extent of ENM transformation in the environment, we present a comprehensive and mechanistic structural investigation of the transformation, aggregation, and dissolution behavior of a polyvinylpyrrolidone-coated silver nanoparticle (AgNP) suspension upon sulfidation in moderately reduced hard water with fulvic acid and dissolved Na_2_S. This reaction is among the most prevalent and industrially and environmentally relevant ENMs transformation. Using *ex situ* transmission electron microscopy (TEM) and both *in situ* and *ex situ* synchrotron-based small angle X-ray scattering (SAXS) and X-ray diffraction (XRD), we find that sulfidation of faceted AgNPs strongly depends on the crystallographic orientation of the facets, with nanometer-scale passivation layers developed on {111} and {100} facets and continuous nucleation and growth on {110} facets. Nanobeam electron diffraction and atomic resolution imaging show Ag and Ag_2_S domains both possess a high degree of crystalline order, contradicting amorphous structures as previously reported. *In situ* SAXS/XRD allowed simultaneous determination of the morphological changes and extent of sulfidation of AgNPs. SAXS/XRD results strongly indicate sulfidation follows first-order reaction kinetics without any aggregation. Aided by their size monodispersity, for the first time, using direct, *in situ* morphology and atomic-structure probes whose results mutually corroborate, we unequivocally determined the sulfidation rate constant of AgNPs under an environmentally relevant condition (≈0.013 min^−1^ for 68 nm diameter AgNPs). A rigorous analysis of the long-term sulfidation product of the AgNPs under different S/Ag ratios using *ex situ* SAXS/XRD clearly demonstrates that the silver mass in the original AgNP and transformed Ag/Ag_2_S NP is preserved. This result has important environmental implications, strongly suggesting that Ag^+^ ions, a known highly effective antimicrobial agent, are not leached into the solution during sulfidation of AgNPs. The combined nondestructive methodology can be extended to unfold the structure transformation pathway and kinetics in a broad range of ENM systems.

## Abstract 2

Silver sulfidation under environmentally relevant conditions follows a first-order reaction kinetics without aggregation or dissolution. Rate and extent of sulfidation demonstrates strong dependence on the crystallographic orientation of the facets.

## Graphical abstract

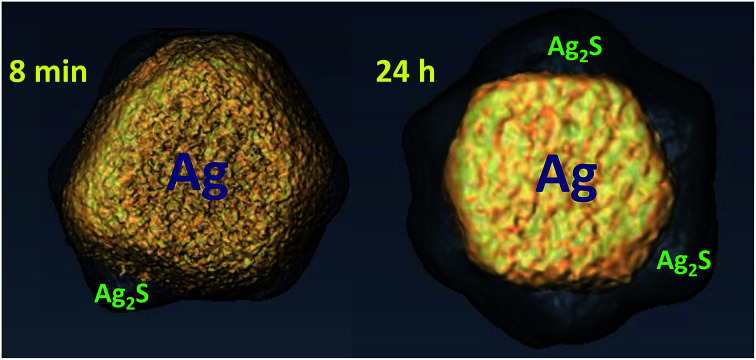

## Introduction

Due to their novel physical and chemical properties, engineered nanomaterials (ENMs) have found increasing applications in medicine,^1^ energy,^2^ sensor technologies,^3^ and consumer industries.^4^ Once deployed, ENMs are subject to their working environments, where ENM structural transformation often occurs.^5^ A proper assessment of the efficacy, safety, and environmental impact of ENMs requires an understanding of the transformation pathway of these materials.^6^ ENM transformation and its related kinetics, however, have only been explored very limitedly, largely due to the complexity arisen from concurrent transformations at different length scales *in situ*. A rigorous and comprehensive determination of ENM transformation in a mechanistic way requires not only advanced materials characterization tools but also in-depth knowledge of the materials system to allow proper modeling of complex data. This lack of understanding presents a major challenge in fulfilling the promises of these novel and attractive materials.

ENM transformation comes in the form of chemical transformations such as oxidation, sulfidation, or reduction reactions, physical transformations such as aggregation and agglomeration, and biologically and environmentally mediated transformations such as surface adsorption of macromolecular ligands or ions.^7–12^ This vast parameter space makes elucidation of ENM transformation difficult. Nevertheless, to predict ENM performance and environmental impacts, knowledge of the extent and rate of specific transformations must be acquired.

Due to their antimicrobial capabilities, silver nanoparticles (AgNPs) are among the most widely used ENMs.^13^ For ENMs, one central concern is their environmental impact and consequent social cost.^6^ For AgNPs in particular, while they can effectively release silver ions in targeted applications, they can also pose significant risks to the ecosystem and overall environment.^14–16^ Thus, it is important to characterize and understand AgNP transformation and its kinetics in realistic environmental settings to elucidate their toxicological behavior. Once discharged into the environment, AgNPs are particularly subject to physiochemical interactions with natural organic matter (NOM), especially humic substances, which act to modify the stability and mobility of AgNPs through electrosteric interactions or hydrophobic effects.^17–19^ Consequently, humic substances influence the stability, dissolution, and aggregation behaviors of AgNPs and affect their transport properties and environmental persistence.^20–22^

To determine sulfidation kinetics of AgNPs, most existing studies relied on proxy measurements such as ion selective electrodes and colorimetric analysis to measure the transformation rates and to infer the mechanism.^23–25^ A separate study of ours strongly suggests that results from such indirect measurements alone can be inadequate and even misleading and that direct measurements that monitor the chemical changes of the metallic NPs are necessary to elucidate the underlying structure transformation.^26^

From the structure point of view, the phase and morphology of AgNPs transform in the environment. The transformation pathway has been extensively investigated in both lab-based and realistic environment settings.^25,27–32^ However, a unified picture of AgNP transformation pathway is yet to emerge. An accurate determination of the kinetics remains elusive. On the one hand, experimental findings from different studies often present contradicting results, preventing general conclusions from being drawn and making it difficult, if not impossible, to establish thermodynamic models with high predictability. On the other hand, it has been considered that “the large number of permutations of nanomaterials and environmental systems makes (comprehensive individual-based case studies) impossible in practice”.^5^ Hence, simplified yet controlled studies of ENM transformations in representative environments may be best positioned to unveil the underlying transformational mechanisms.

Meanwhile, the characterization of structural transformations in ENMs presents a well-documented fundamental challenge.^5,24,28^ While various *ex situ* analytical techniques play a central role, they usually focus on the final transformation product and do not reveal transient states. Thus, *ex situ* methods often fail to directly capture the rate and extent of the transformations, an issue so significant that a recent Consensus Study Report from the Unites States National Academies^33^ identifies *in situ* and *in vivo* methods to determine the potential and rate of fundamental ENM transformation processes as an urgent research priority.

One key aspect of the characterization challenge is that structural transformations of ENMs generally occur across a broad range of length scales. For example, chemical transformation and dissolution of ENMs often occur at the atomic level whereas aggregation and agglomeration occur at the nano- and micro-meter scales. To overcome this challenge, various synchrotron-based *in situ* X-ray techniques have been developed recently to probe nanoparticle synthesis, growth, and transformation in a liquid environment. In particular, one representative work published in *Science* in 2017 by Sun *et al.* constitutes one of the first papers that detail the ENM transformations in real time (oxidation process of colloidal Fe–Fe_*x*_O_*y*_ NPs).^34^

Similarly, to offer a possible solution to this metrological challenge, with the overarching goal of a more solid understanding of the structural transformational pathway of ENMs, we have conducted a series of studies of AgNPs using *ex situ* transmission electron microscopy (TEM) and both *in situ* and *ex situ* synchrotron-based ultra-small angle X-ray scattering (USAXS), small-angle X-ray scattering (SAXS) and X-ray diffraction (XRD). TEM presents direct visual evidence of the morphology and extent of the transformation of individual ENMs. *In situ* X-ray studies allow characterization of the rate and extent of the transformation on a statistically significant basis. Importantly, when USAXS, SAXS, and XRD are combined, they encompass a continuous length-scale range from sub-angstrom to several micrometers,^35–38^ which allows both chemical and physical transformation of ENMs to be determined simultaneously. *Ex situ* X-ray studies allow the crystallinity and extent of the structure transformation of the end-product to be understood comprehensively. Comparing with the techniques used in Sun *et al.*,^34^ our approach has the added advantage of being able to unambiguously determine the aggregation state of the AgNPs, due to the broader accessible *q* range of USAXS.^39^ When used together, these techniques provide a window to peer into the intricate transformational kinetics of ENMs.

Our study is conducted in a controlled model system, where sulfidation is investigated for monodisperse, polyvinylpyrrolidone (PVP) coated AgNPs suspended in water with NOM (Suwannee River fulvic acid). ENM reactivity is strongly related to particle size.^40^ The narrow size distribution of AgNPs used in this study allows the sulfidation transformation process to be differentiated from other processes, quantitatively characterized, and the transformation rate to be established precisely. Sulfidation is the key environmental structural transformation of interest for AgNPs.^41,42^ The transformation of AgNPs in the presence of NOM is of intense current research interest because of the pervasiveness of ENM interactions with NOM in realistic environmental settings. We hope that our controlled study provides insights into the specific structural transformations and their kinetics associated with sulfidation of AgNPs in increasingly complex and realistic environmental settings, and more generally, helps establish a methodology that determines the transformation rate and potential of ENMs.

## Materials and methods

### Starting materials‡‡Certain commercial equipment, instruments, software or materials are identified in this paper to foster understanding. Such identification does not imply recommendation or endorsement by the Department of Commerce or the National Institute of Standards and Technology, nor does it imply that the materials or equipment identified are necessarily the best available for the purpose.

The AgNPs are derived from NIST Reference Material (RM 8017, NIST, Gaithersburg MD), with a nominal core diameter of 75 nm and coated with PVP of average molecular weight of 40 kDa.^43^ Sodium sulfide nonahydrate (≥99.99% trace metal basis) was acquired from Sigma-Aldrich (St. Louis, MO) and used without further treatment. We acquired Suwannee River Fulvic Acid Standard I (FA) from the International Humic Substances Society (St. Paul, MN).^44^ The moderately hard reconstituted water (MHRW) solution was prepared following a protocol established by the U.S. Environmental Protection Agency.^45^

### TEM measurements

TEM images were acquired using a probe-corrected FEI Titan transmission electron microscope operated at 300 kV. High-angle annular dark field scanning TEM (HAADF-STEM) images were acquired using a Fischione Model 3000 detector and the inner collection angle was set to ≈71 mrad. Electron energy loss spectroscopy (EELS) data were collected with an outer collection angle of ≈11 mrad. For EELS and HAADF-STEM a convergence angle of ≈13.5 mrad was used and the probe current was typically 20 pA to 30 pA. Nanobeam electron diffraction (NBED) patterns were collected with a semi convergence angle of ≈1 mrad. Tomography datasets were acquired in 3° steps. The probe convergence angle was ≈9 mrad and a detector inner collection semi-angle of ≈42 mrad or ≈58 mrad was used. The probe current was ≈10 pA. The tilt series data was aligned and reconstructed using Inspect 3D and OpenMBIR,^46^ respectively. Data visualization was performed using Avizo.

The lyophilized AgNP RM containing 2 mg of Ag and 20 mg of PVP was reconstituted by redispersion in 2 mL of deionized water. The resulting suspension was purified by centrifugal ultrafiltration using Amicon Ultra-4 centrifugal filter units with nominal molecular weight limit of 100 kDa (EMD Millipore, MA). Mixture of FA solution and MHRW was pH adjusted using NaOH to 7.0 ± 0.2. AgNPs were added to the mixture. Mixing of AgNPs was achieved by manual shaking for 10 s. Freshly prepared Na_2_S solution was finally introduced to avoid potential interaction between the sulfide and NOM.^29^ In the final stock suspension, the mass concentration of AgNPs was 1.62 mg L^−1^, and the mass ratio between FA and Ag and the molar ratio between S and Ag were 5.0 and 0.72, respectively. TEM aliquots were taken at 8 min, 30 min, 1 h, 8 h, and 24 h after Na_2_S was introduced. Purified samples were deposited onto Ni grids with a carbon support film stored in a vacuum box and examined typically within 2 days of preparing the grid.

### Synchrotron measurements

Synchrotron USAXS, SAXS, and XRD experiments were performed at the USAXS facility at the Advanced Photon Source (APS), Argonne National Laboratory.^47,48^ The X-ray wavelength was 0.05904 nm. The absolutely-calibrated USAXS measurements were conducted using the instrument's standard 1-D collimated geometry.^49^ The SAXS and XRD experiments were conducted using two standalone Pilatus 2-D area detectors (Model: 100K-S, Dectris, Baden, Switzerland).^50^ The data acquisition times for USAXS, SAXS, and XRD were 90 s, 30 s, and 30 s, respectively.

The *in situ* measurements were conducted with a continuous flow of the sample suspension through a custom-made flow cell, following the steps below:

(1) 1.5 mL of pH adjusted (pH 7) FA solution (64.86 mg FA was dissolved in 0.7 mL water and 0.8 mL MHRW) was added into 6 mL of purified AgNP suspension (Ag concentration 2.16 mg mL^−1^) in a vial to achieve a mass ratio for FA to AgNPs of 5 : 1. After vigorous shaking for 1 min, the combined USAXS/SAXS/XRD dataset was collected as the baseline of the pristine state of the AgNP suspension.

(2) 0.022 g crystalline Na_2_S·9H_2_O was dissolved in 0.5 mL of DI water. AgNP concentration after the addition of Na_2_S was 1.62 mg mL^−1^. *In situ* experiments were started after the Na_2_S solution was added to the AgNP suspension by conducting a repeated sequence of USAXS, SAXS, and XRD measurements. Each set of USAXS/SAXS/XRD measurements took ≈5 min.

The *ex situ* samples were prepared approximately 10 days before the synchrotron measurements following a similar protocol to that of the *in situ* sample with the same starting materials. The main difference with the *ex situ* samples was that the molar ratio between S and Ag was adjusted systematically from 0 to 5. Details of these samples can be found in Table 1. The *ex situ* measurements were conducted using standard liquid cells available at the beamline. Necessary scattering data correction steps with liquid cells are described elsewhere.^51^

**Table 1:** Details of the *ex situ* samples reported in this study, including the concentrations of AgNP, FA, and S. All samples were prepared in MHRW at pH 7

Sample identifier	[AgNPs] (mg mL^−1^)	[FA] (mg mL^−1^)	*n*S/*n*Ag
ES0	2	10	0
ES1	2	10	0.1
ES2	2	10	0.3
ES3	2	10	0.5
ES4	2	10	1
ES5	2	10	5

More details about the synchrotron measurements can be found in the ESI.†

## Results and discussion

### *Ex situ* TEM characterization

TEM provides direct visualization of nanoparticle structure and morphology, and has been used extensively to determine the fate of AgNPs upon environmental exposure.^27,29,30,52–54^ In this study, we used a suite of TEM-based analytical techniques to characterize the morphology, atomic structure, and elemental distribution of individual AgNPs at different stages in the sulfidation process.

The pristine AgNP specimens showed no aggregation under TEM.^43^ Analysis of 96 particles showed a particle diameter of 67.5 ± 5.1 nm. The AgNPs were in the form of polyhedrons predominately with {111} and {100} surface terminations. The vertices of the particle were nominally {110} terminated, and the particle edges often had a rounded appearance resulting from surface steps and higher-order surface terminations, a feature identified earlier in a silver cube nanoparticle system.^55^ High-resolution images and an atomistic model of pristine AgNPs illustrating the most commonly observed AgNP geometry are shown in ESI.†

**Fig. 1:**
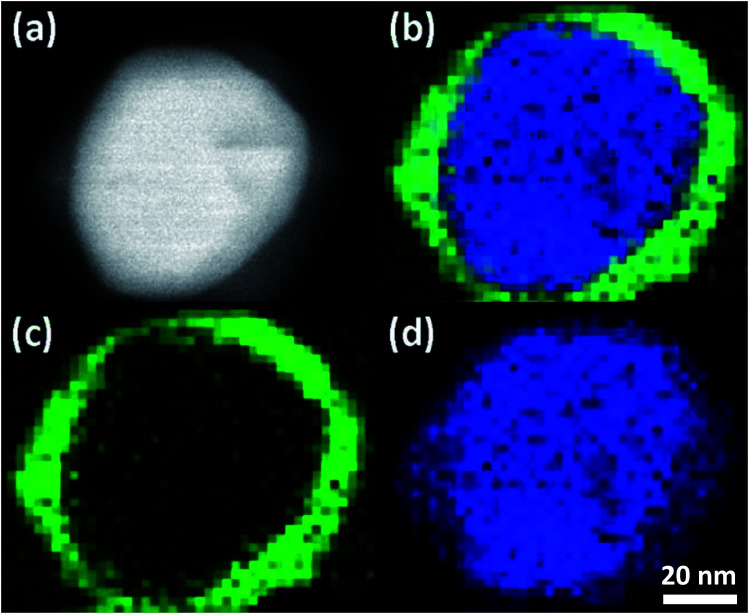
The contrast of the HAADF image of a Ag_2_S/Ag particles after 1 h (a) is sensitive to the atomic number difference between the Ag_2_S and Ag domains. (b) Colorized EELS spectrum images from the same particle where green is sulfur (c) and silver is blue (d).

Chemical mapping by STEM-EELS identifies the spatial distribution of elements within the reacted AgNPs. An example of colorized elemental maps from AgNP reacted for 1 h is shown in Fig. 1. The composite image in Fig. 1(b) shows that the reacted AgNP is composed of Ag and S. The distribution of Ag and S, however, is not uniform. As shown by Fig. 1(c) and (d), S is enriched near the surface, while Ag is identified in all parts of AgNP. The HAADF image in Fig. 1(a) reflects this compositional inhomogeneity as the image contrast of this technique is sensitive to atomic number. As reported previously,^29^ when pH ≥ 7, Ag binds strongly with S in natural system following a direct conversion: 4Ag + 2HS^−^ + O_2_ → 2Ag_2_S + 2OH^−^. The STEM-EELS result suggests initially sulfidation is dominated by a surface reaction between Ag and S with the silver core intact, suggesting a direct exchange mechanism rather than a vacancy exchange mechanism observed in the Kirkendall effect.^25^

**Fig. 2:**
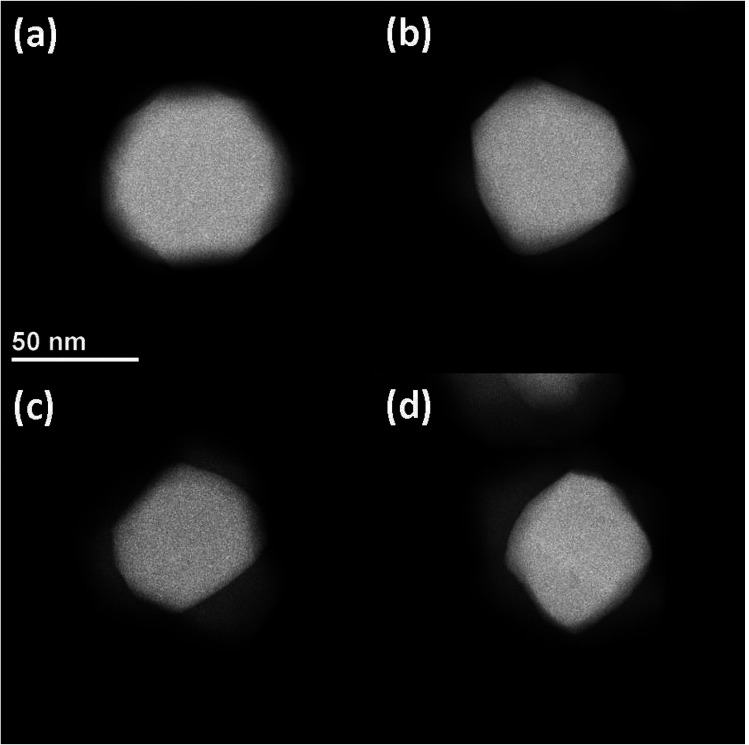
Representative examples of Ag_2_S/Ag particles at 8 min (a), 1 h (b), 8 h (c) and 24 h (d) of reaction time. The brighter central core is the Ag while the less intense regions nucleating along the particle vertices are Ag_2_S. All particles are [111] oriented and the magnification is the same (see scale bar).

The atomic number contrast provided by HAADF-STEM can be used to track the extent of the structural transformation from Ag to Ag_2_S. Typical TEM images of aliquots taken at 8 min, 1 h, 8 h, and 24 h after Na_2_S was introduced are shown in Fig. 2. Here all the AgNPs were 〈111〉 oriented to facilitate intuitive comparison. The reacted nanoparticles contained a bright core and less bright regions growing at particle vertices along the {110} terminations, as shown in Fig. 2(a). With increasing reaction time, the overall particle size increased slightly, the relative volume of the Ag cores decreased, and the relative volume of the Ag_2_S domains increased. The growth of the Ag_2_S domains eventually led to their impinging on one another (Fig. 2(b–d)). After 24 h, unreacted silver core was clearly visible, indicating incomplete conversion. More HAADF-STEM data, as well as tomographic reconstructions of two AgNPs sulfidized for 8 m and 24 h, can be found in Fig. S3–S8 in ESI and the Movies.†

**Fig. 3:**
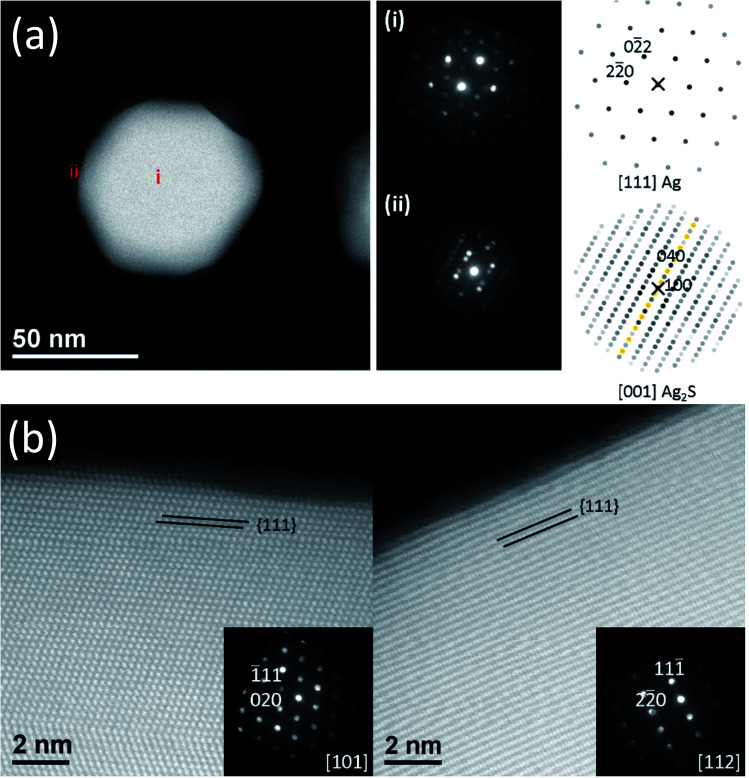
(a) NBED diffraction patterns from a Ag_2_S/Ag particle after 8 min identify the phase as (i) Ag (space group 225, *Fm*3̄*m*) and (ii) Ag_2_S (space group 14, *P*2_1_/*c*). Note in (i) forbidden reflections are present due to planar defects. The NBED patterns have been rotated to correspond to the orientation of the real space image and a nonlinear histogram adjustment was made to highlight the presence of weak features. Simulated diffraction patterns accompany the NBED patterns. (b) TEM Images of passivated surfaces. {111} surfaces after 24 h have only a thin Ag_2_S layer present. NBED patterns (inset) have been rotated to correspond to the orientation of the real space image and a nonlinear histogram adjustment was made to highlight the presence of weak features.

We used NBED to examine the degree of crystallinity of the Ag_2_S domains and Ag core during the structural transformation. Typical diffraction patterns are shown in Fig. 3(a). Both Ag core and Ag_2_S domains are crystalline. Ag has a structure of *Fm*3̄*m* with lattice parameter *a* = 0.409 nm. Ag_2_S has a structure of *P*2_1_/*n* with *a* = 0.423 nm, *b* = 0.691 nm, *c* = 0.787 nm, *α* = 90°, *β* = 99.58°, and *γ* = 90°. More NBED data are shown in Fig. S9.† It is worth noting that at all reaction intervals, our results show Ag core and Ag_2_S domain were fully crystalline, a result in contradiction to some reports in literature.^25,32^ For example, Levard *et al.* found that without NOMs, with the S/Ag ratio in the range of 0.019 and 0.719, PVP-coated AgNPs transformed to amorphous Ag_2_S.^32^ NOMs are known to affect colloidal stability and dissolution of AgNPs. Our results infer that NOMs may also regulate the atomic-scale structure transformation during silver sulfidation.

Interestingly, our results demonstrate that the sulfidation process is sensitive to the faceting of the Ag surface, as shown in Fig. 3(b). The {111} surfaces at the top and bottom of the AgNPs remained passivated for all aliquots examined (up to 24 h). Amorphous passivation layers formed with nm layer thickness. It was not possible to conclusively confirm the phase of this passivation layer. However, based on the HAADF image contrast and strong reactivity between Ag and S makes Ag_2_S most likely. This result is in good agreement with a recent study of Ag–Ag_2_S triangular hybrid nanoprisms by Mirkin *et al.*, where a thin passivation layer of Ag_2_S on the Ag {111} facets was indirectly observed.^56^ The {110} terminated vertices, on the other hand, did not passivate, which allow Ag_2_S nucleation and growth. {100} and {111} surfaces located at the AgNP sides were not observed to function as separates sites for the nucleation and growth of Ag_2_S, but were eventually transformed to Ag_2_S as the reaction front proceeds inwards from the tips towards the core. The conversion, starting from the vertices, proceeds macroscopically along the 〈110〉 directions, however the atomic level mechanism of the reaction front appears to be the collective response of the transformation occurring along of multiple crystal planes as illustrated from slices of a tomographic reconstruction shown in Fig. S10.† The dependence of reactivity of Ag surface on its crystallographic orientation was known for bulk Ag, where it was shown that the formation of Ag_2_S adlayer can only occur without significant reconstruction of the outermost atomic layer of the substrate.^57^ For nanosilver, however, the reaction energetics can be further complicated by geometrical effects – facets at the tip may have energetically unfavorable atomic structures that lead to higher reactivity, which may contribute to our observation of sulfidation progression from the vertices of the AgNPs, an observation also made by others.^58,59^ Elucidation of the reactivity will require density functional theory calculations, and is out of scope of this paper. Nevertheless, our observation of different reactivity along different crystallographic orientations is clear.

### *In situ* SAXS/XRD

Synchrotron-based SAXS and XRD, as an analytical tool, can reveal kinetics associated with nanoparticle transformation, aggregation, and agglomeration.^60^ In our *in situ* SAXS/XRD study, we used SAXS to investigate the morphological transformation kinetics of the AgNPs, and XRD to investigate the structural transformation of the AgNPs, acquiring complimentary structural information across a sub-nanometer to micrometer length scale.

**Fig. 4:**
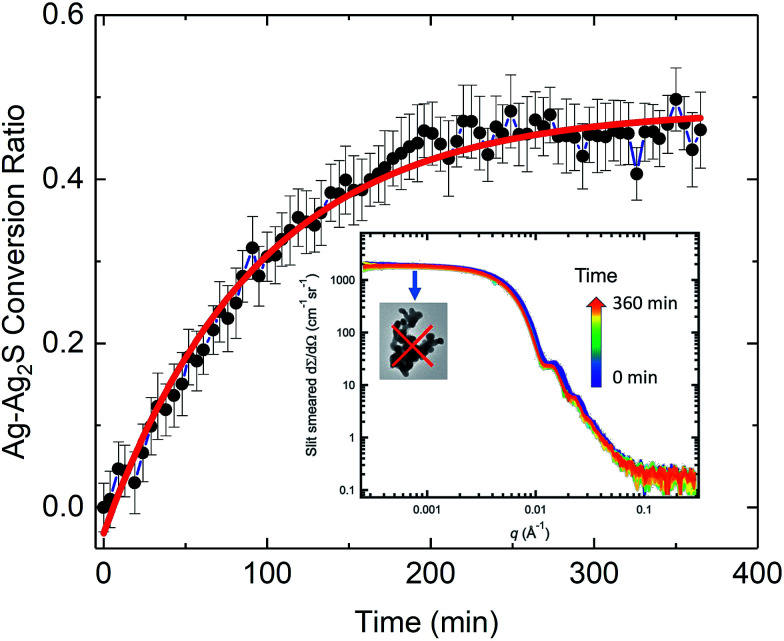
Time-dependent conversion ratio of Ag to Ag_2_S. The solid line represents a least-squares fit using a pseudo-first order rate model in the form of an exponential decay function. The inset shows time-dependent evolution of the SAXS profiles upon initiation of the sulfidation reaction. A total of 80 scattering curves are plotted on a log–log scale in the figure, which spans a time of over 6 h. Time of acquisition is illustrated by the color bar. The highlighted low-*q* plateau clearly shows that AgNP aggregation did not occur in the duration of the *in situ* SAXS/XRD study. Here and in subsequent figures, vertical bars on data points represent computed standard deviation uncertainties.

Time-dependent SAXS data are shown in the inset of Fig. 4, with acquisition time indicated by a color scale. As time increases, the scattering curves shift to smaller *q*, indicating a gradual increase of particle size, consistent with TEM observations, which show growth of Ag_2_S domains along the {110} facets. The growth of Ag_2_S domains is also supported by the change in color of the AgNP suspension during the *in situ* study. The color of the initial AgNP suspension was gray. Soon after the introduction of Na_2_S solution, we observed the suspension color changed to, and remained, black until the end of the measurements, which is consistent with reported optical properties of Ag_2_S.^28^ The Bessel oscillations in the scattering curves persisted throughout the duration of the measurements, indicating that a narrow particle-size distribution was maintained. A distinct plateau is always identifiable in the low-*q* regime of the scattering curves, showing that the AgNPs did not form aggregates (*i.e.*, they did not coalesce) as they were being sulfidized and the particles remained well dispersed.^36^ Thus, the retained colloidal stability of the AgNPs in suspension during the sulfidation process under the conditions measured is conclusively established.

We analyzed the time-dependent evolution of the mean particle size assuming that the particle volume-size distribution follows a Gaussian form using the SAXS analysis package, Irena.^61^ As we show later, it can be established that, under our experimental conditions, the total mass of Ag in the transformed Ag/Ag_2_S nanoparticles is preserved during the sulfidation process. Treating this simply as an assumption here, we derived the conversion ratio, defined as the ratio of Ag mass in reacted Ag_2_S product within any one nanoparticle to its starting pristine (pure Ag metal) Ag mass value, from the mean size of the particles. Details of the SAXS analysis are provided in the ESI.†

For pristine (unreacted) AgNPs, we found that the particle diameter with standard uncertainty is (68.6 ± 6.4) nm. Because *in situ* SAXS experiments characterized ≈1.5 × 10^8^ AgNPs at one time, this result is statistically-representative and confirmed that the AgNPs had a very narrow size distribution. It is known that the size of nanoparticles is strongly tied to their activation energy and reaction rate constant.^62^ For kinetic rate determination of the AgNP transformation, a goal of the current *in situ* study, we emphasize that the identified monodispersity of the pristine AgNPs is important, and we recommend that the size monodispersity be carefully controlled in future rate studies. Additionally, we point out that X-rays are sensitive to high-Z elements because of their high X-ray scattering-length density. The SAXS NP diameters measured concern the physical dimensions of the AgNPs and the subsequent reacted Ag/Ag_2_S NPs only, but they provide no direct information regarding the PVP coating and presence of NOM materials near the surface of the NPs.^36^

Fig. 4 shows the time-dependent conversion ratio of Ag to Ag_2_S, a transformation conclusively demonstrated by TEM. Hence, this conversion curve is directly related to the sulfidation kinetics of AgNPs. The conversion was rapid initially, then gradually slowed down, approaching a plateau. We analyzed the kinetic rate using a pseudo-first order rate model (in the form of exponential decay), similar to one previously used to describe the sulfidation kinetics of AgNPs.^29^ We found that the rate constant is (0.0107 ± 0.0005) min^−1^. Interestingly, this value is smaller than the kinetic rate identified for 30 nm nanoparticles in Liu *et al.*,^29^ where the sulfidation kinetics is deduced from the time-resolved depletion of sulfide. While it might be tempting to conclude that the larger specific area of smaller AgNPs leads to a faster sulfidation, important differences between these two experiments must be noted. In contrast to probing the sulfidation of AgNP powder with no coating by Na_2_S in water, our experimental conditions approximate more closely to a realistic environmental setting, where factors such as the presence of NOM, surface functionality, as wells as particle size, can all affect the rate of transformation kinetics. Such differences point to the challenges in predictive modeling, where complexity due to a large set of parameters must be expected.^63^

Our TEM and SAXS results unequivocally demonstrated that under our experimental conditions, AgNPs did not aggregate after 24 h of reaction. We also conducted further studies, where we investigated the role of pH, the presence of fulvic acid, and the type of humic substance, on the colloidal stability during sulfidation of the same type of AgNP suspension in both water and MHRW. These results, to be reported elsewhere, again show consistent colloidal stability of the AgNPs over a long period of time (hours to days). This consistent colloidal stability contrasts with some of the existing studies of AgNPs in real and simulated environmental systems, where aggregation behaviors were observed for AgNPs.^24,27,31,64,65^ Furthermore, while recent work has suggested that the presence of humic substances may aid the colloidal stability of AgNPs,^29,66,67^ Zhu *et al.* reported that humic acid modified the surface coverage of PVP *via* adsorption or ligand exchange and sulfidation removed PVP from the particle surface and consequently reduced the colloidal stability of AgNPs.^20^ This wide spectrum of reported results is not surprising. The colloidal stability of nanoparticles requires a delicate balance between forces such as van der Waals attraction, steric repulsion, coulombic interaction, and depletion forces. In ENMs, it is often the surface ligand and coating that plays a central role in controlling their colloidal stability and aggregation state.^40^ Our results, as additional evidence, invite a systematic investigation of the detailed role of sterically protecting polymers and NOMs on the colloidal stability of model AgNP systems during sulfidation, an essential component of ENM processing and application.^40^

**Fig. 5:**
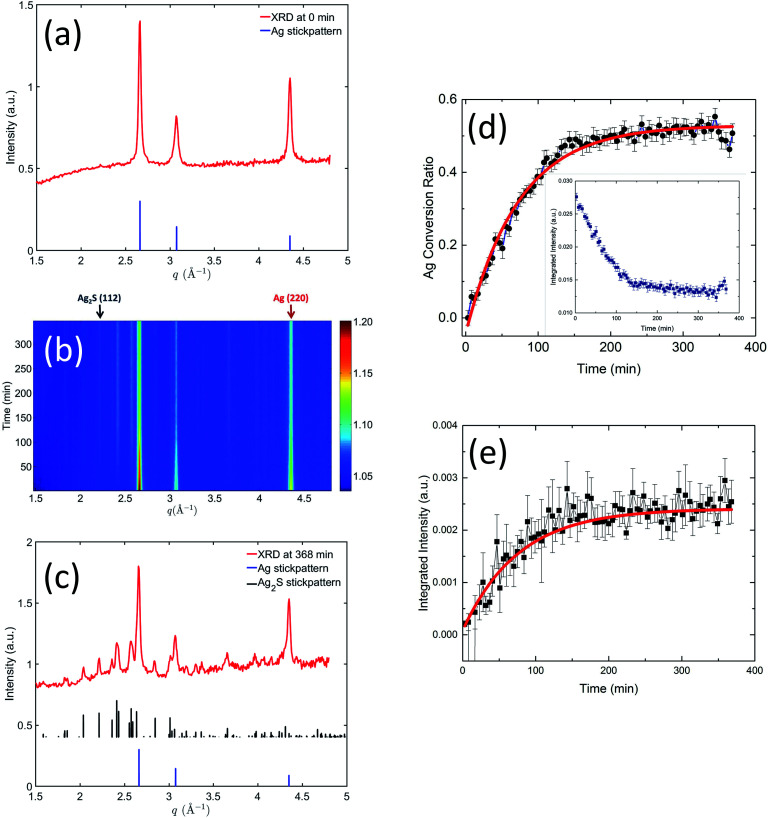
(a) XRD pattern recorded from pristine AgNP suspension. (b) Two-dimensional contour plot of the *in situ* XRD patterns showing the time-dependent evolution of crystalline phases present during sulfidation. (c) XRD pattern recorded from AgNP suspension at 368 min after the sulfidation process was initiated. The reference stick patterns were simulated using the space groups and lattice parameters shown in the TEM section. (d) shows the conversion ratio of Ag to Ag_2_S, based on the integrated intensity of Ag (220) peak shown in its inset. (e) shows time-dependent evolution of the integrated intensity of Ag_2_S (112) peak. In (d) and (e), the solid lines represent least-squares fits of the kinetics data using an exponential decay function.

While SAXS probes the physical morphological transformation of the AgNPs, XRD, being a diffraction technique, provides structural fingerprints of the phases present and their evolution. We devised a XRD data reduction procedure for weak diffraction intensity of ENMs in solution, documented in the ESI.†Fig. 5 shows the time-resolved XRD results, which illustrate in real time the variation of crystalline phases of the AgNPs. Initially (Fig. 5(a)), the pristine AgNPs were single-phase silver, demonstrated by the diffraction data perfectly matching the simulated Ag XRD reference stick pattern (the reference stick patterns here and hereinafter were simulated using the space group and lattice parameters identified in the TEM section). Fig. 5(b) presents a two-dimensional contour plot of the *in situ* XRD patterns recorded at different times during the sulfidation process. It is evident that with increasing reaction time (from bottom to top), the primary silver peak intensities decreased, and concurrently a family of weak diffraction peaks emerged with increasing intensity, indicating a gradual structure transformation. Fig. 5(c) shows the XRD pattern acquired at 368 min into the reaction. A comparison with the XRD reference stick patterns of Ag and Ag_2_S clearly shows the presence of Ag_2_S, again proving Ag was transformed to crystalline Ag_2_S. Ag XRD peaks persisted at 368 min, albeit at a lower intensity compared with their counterparts in the pristine state, a result that is in good agreement with the TEM findings.

We performed quantitative analyses on the peak profiles of two stand-alone peaks: the Ag_2_S (112) peak and the Ag (220) peak, as highlighted in Fig. 5(b), to investigate the crystalline transformation kinetics. These results are shown in Fig. 5(d) and (e). Here, we normalized the integrated peak intensity of Ag (220) peak to that of the pristine Ag, translating XRD peak intensity to the molar ratio of Ag transformed to Ag_2_S. We performed a least-squares analysis on the intensity evolution of these two peaks using the same exponential decay model as in the SAXS kinetics analysis. The acquired rate constants from the XRD analysis are summarized in Table 2. The rate constants acquired from the declining Ag (220) peak and the increasing Ag_2_S (112) peak are equivalent within the uncertainties, which suggests that Ag transformed to Ag_2_S without significant dissolution. Furthermore, a comparison of the SAXS and XRD kinetic time scales shows that they are similar, indicating that both SAXS and XRD probed fundamentally the same process, *i.e.*, the increase in particle morphology (size) is directly related to the chemical transformation from Ag to less dense Ag_2_S.

**Table 2:** Kinetic rate and time scales acquired from morphological analysis of the AgNPs and the peak profiles analyses of Ag (220) peak and Ag_2_S (112) peak

	Rate constant (min^−1^)
USAXS/SAXS	0.0107 ± 0.0005
XRD, Ag (220) peak	0.0138 ± 0.0005
XRD, Ag_2_S (112) peak	0.0132 ± 0.0012

Notably, at the end of the *in situ* experiment, SAXS and XRD results demonstrate remarkable consistency and pointed to the same conversion ratio of Ag to Ag_2_S (SAXS: 0.46 ± 0.04, XRD: 0.50 ± 0.05). Interestingly, despite an abundance of sulfide ions during the *in situ* experiment, only 50% of Ag was transformed. The reason for this is unclear. It is known that sulfide depletion can occur when humic substances are present, even without AgNPs.^29^ We speculate that possible sulfide-NOM complexing may reduce the availability of sulfide during this initial stage of sulfidation.

### *Ex situ* SAXS/XRD

While the *in situ* SAXS/XRD experiments provide insights into the kinetic rate of the AgNP transformation during sulfidation, they nevertheless cannot capture the entire transition pathway due to limitations imposed by beam time availability. To understand the impact that the molar S/Ag ratio has on the structure and morphology of the end-product, we conducted *ex situ* SAXS/XRD measurements on samples that had been subject to sulfidation at different S/Ag ratios for approximately 10 days. We note that at this AgNP concentration and pH, the oxidation rate of AgNPs is very slow. Repeated single-particle inductively coupled plasma mass spectrometry (ICP-MS) measurements of 1 mg mL^−1^ AgNP suspensions did not show any significant change in particle size over >200 days. Hence, we can assume the change in the particle morphology and crystal structure is due to the sulfidation reaction, alone.

**Fig. 6:**
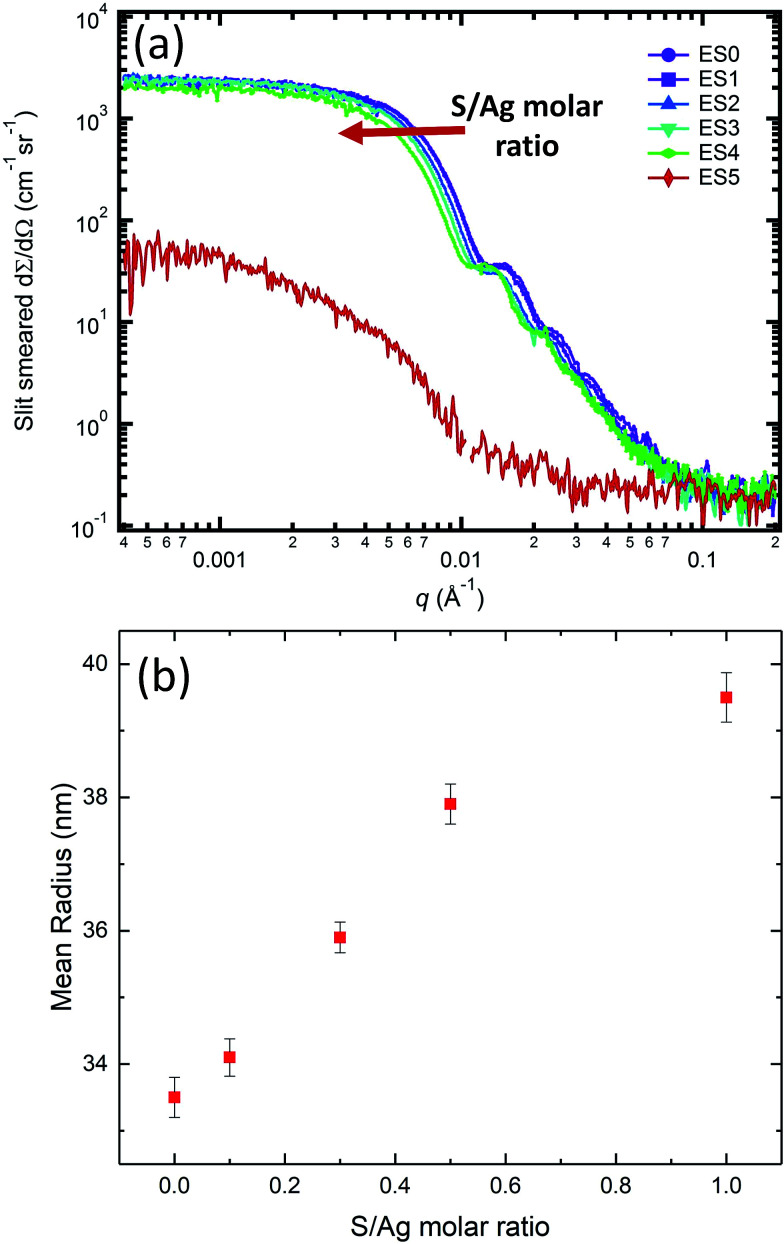
(a) Scattering profiles of the *ex situ* AgNP suspension samples with S/Ag ratio of 0 (EB0), 0.1 (EB1), 0.3 (EB2), 0.5 (EB3), 1.0 (EB4), and 5.0 (EB5), respectively. (b) The dependence of mean size of the *ex situ* AgNP samples EB0-EB4 on the S/Ag molar ratio.

Fig. 6 presents the SAXS results for the *ex situ* samples listed in Table 1. When the S/Ag molar ratio was between 0 and 1, the colloidal stability of AgNPs was maintained, and the narrow size distribution and the overall particle morphology were preserved as evidenced by the continued presence of the Bessel oscillations. However, at S/Ag = 5, such observations were no longer valid. Here, we observed scattering signatures from aggregates, as well as visible sedimentation, leading to a significant decrease of the scattering intensity as *q* → 0. For S/Ag ≤ 1, the mean radius increased monotonically with increasing S/Ag ratio (Fig. 5(b)), indicating that AgNP sulfidation progressed in accordance with the total amount of available sulfide in the starting solution.

**Fig. 7:**
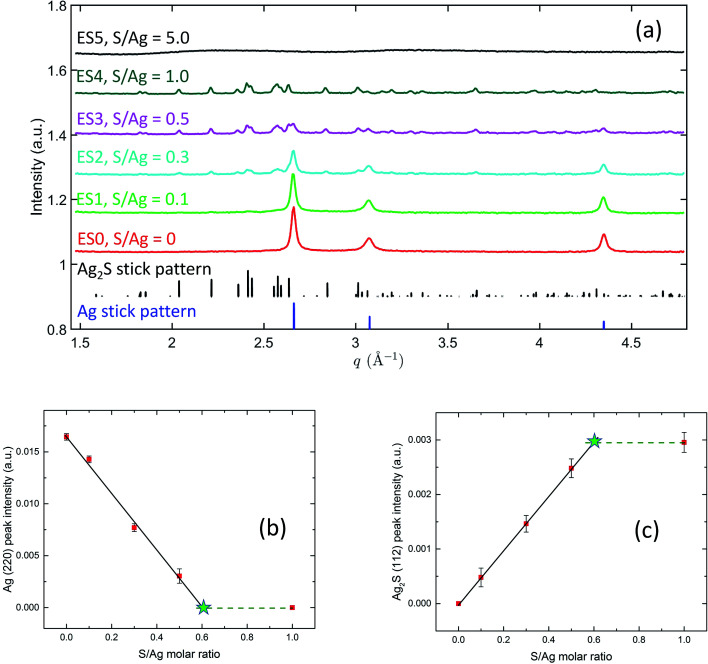
(a) XRD patterns recorded from *ex situ* AgNP suspension samples. The reference stick patterns were simulated using the space group and lattice parameters shown in the TEM section. (b) and (c) show the dependence of the integrated intensity on S/Ag molar ratio of Ag (220) peak and Ag_2_S (112) peak, respectively. The solid lines represent fits from a linear least-squares regression analysis. The dashed lines are for viewing purpose only. The stars, located at the intersects between the solid lines and dashed lines, show the values of S/Ag ratio at which full transition from Ag to Ag_2_S occurs.

Fig. 7(a) captures the phases present in the *ex situ* AgNP samples after sulfidation reactions have occurred with different S/Ag ratios. A comparison with the Ag and Ag_2_S reference stick patterns shows that, on increasing the S/Ag ratio, the Ag peak intensities monotonically decreased and Ag_2_S peak intensities monotonically increased. This reveals a systematic transformation from Ag to Ag_2_S depending on the availability of sulfide in the solution. It is worth highlighting that at S/Ag = 1, the characteristic Ag diffraction peaks disappeared altogether. With the high sensitivity of the synchrotron XRD experiment, this strongly indicates that the transformation from Ag to Ag_2_S was practically complete. At S/Ag = 5, these observations again broke down, with only amorphous diffraction patterns observed. Together with the SAXS observation of nanoparticle aggregation at this S/Ag ratio, these abnormalities suggest that the transformation pathway for Ag sulfidation strongly depends on the availability of sulfide in the solution, with a switchover point between S/Ag = 1 and S/Ag = 5 for both colloidal stability and structural transformation.

The integrated peak intensities of the Ag (220) peak and the Ag_2_S (112) peak are shown in Fig. 7(b) and (c). Notably, in both plots, when S/Ag is between 0 and 0.5, the integrated intensities demonstrate a linear dependence on S/Ag. A linear least-squares regression analysis yields that for Ag, *I*_Ag (220)_ = 0.01665(38) − 0.02828(185) × S/Ag, and for Ag_2_S, *I*_Ag_2_S (112)_ = 0.0006(6) + 0.0048(19) × S/Ag. XRD data at S/Ag = 1 shows the Ag to Ag_2_S transition to be complete. Hence, we can assume that the integrated intensities at S/Ag = 1 represent the terminal intensities, which are plotted as the dashed horizontal lines in Fig. 7(b) and (c). The intersects between the dashed lines and the linear fits, therefore, point to the threshold Ag/S ratios necessary for the full transition from Ag to Ag_2_S to occur. Based on this, we found that for the Ag (220) plot (Fig. 7(b)), the intersect is located at S/Ag = 0.589 ± 0.052, whereas for the Ag_2_S (112) plot (Fig. 7(c)), the intersect is located at S/Ag = 0.604 ± 0.030. This excellent agreement reveals that the full atomic structure transformation requires ≈0.6 S/Ag molar ratio, which is higher than the 0.5 molar ratio that the stoichiometry of Ag_2_S dictates. In the context of the peroxidation of Na_2_S during storage and potential complexing between S and humic substances,^29,68,69^ this may not be completely surprising.

We further deduced the mass of silver within individual nanoparticles in the *ex situ* samples. In particular, data shown in Fig. 7(a) established that with S/Ag = 0, the nanoparticle composition is silver only, and that with S/Ag = 1, the composition is Ag_2_S only. From the SAXS analysis, we determined that the particle radii at S/Ag of 0 and 1 are (33.5 ± 0.3) nm and (39.5 ± 0.4) nm, respectively. With Ag and Ag_2_S densities being 10.49 g cm^−3^ and 7.23 g cm^−3^, respectively, we calculated that the Ag mass per nanoparticle is (1.652 ± 0.015) × 10^−15^ g for S/Ag = 0 sample (ES0) and (1.626 ± 0.017) × 10^−15^ g for the S/Ag = 1 sample (ES4), respectively. The equivalence of these two masses strongly indicates mass preservation of Ag during the sulfidation process. In other words, although oxidation is a necessary step of the sulfidation reaction, when excess S^2−^ is available, Ag^+^ reacts with near-surface sulfide and remains part of the Ag/Ag_2_S nanoparticle. Hence, no Ag is leached to the solution in the form of soluble Ag^+^ ions. This result is consistent with a previous proposal concerning the sulfidation mechanism by Liu *et al.*, where it was suggested that when the concentration of sulfide is high ([sulfide] = 0.025 mg L^−1^), AgNPs directly transform to Ag_2_S without intermediate dissolution and reprecipitation.^29^ The absence of Ag dissolution is critically important because dissolved Ag^+^ ions provide the main basis for the antimicrobial properties of AgNPs and the main cause for environmental concerns associated with AgNPs.^24^ With our analysis, we are able to show that when S/Ag is below the aforementioned unknown threshold value (higher than 1 but less than 5), in the system that we investigated, sulfidation not only reduces the toxicity of AgNPs due to the extremely low solubility of Ag_2_S,^31^ but more importantly, it prohibits soluble Ag^+^ ions from leaching into the solution, thus significantly limiting the environmental impact of AgNPs. It is also worth noting that while the kinetics of AgNP sulfidation may be affected by the surface state of the nanoparticles, previous studies have asserted that the thermodynamics may not strongly depend on the surface coverage of AgNPs due to stability constant considerations.^70,71^ The validity of this assertion can be further tested by *in situ* and *ex situ* studies similar to what is now reported in this work.

## Conclusions

Quantitative understanding of the transformation pathway and its related kinetics of ENMs is a major challenge that impacts the application and certification of these promising materials. Using one of the most prevalent and industrially and environmentally relevant ENMs transformation as an example, in this paper, we have systematically investigated the fundamentally important structural transformation of AgNPs during their sulfidation in water in the presence of natural organic matter. Our methodology involves using a model system where the narrow size distribution of the AgNPs was carefully controlled, a prerequisite for statistically meaningful rate determination due to the well-known strong dependence of nanoparticle reactivity on particle size. Taking advantage of the high quality of the colloidal AgNPs, we applied the synchrotron-based *in situ* USAXS, SAXS, and XRD techniques, which are sensitive to the NP size, morphology, electron density, and phases, to precisely track the sulfidation process of the colloidal AgNPs–Ag/Ag_2_S NPs in real time. By combining rigorous *ex situ* structure determination using analytical TEM, *in situ* and *ex situ* synchrotron SAXS and XRD, we addressed some of the major unanswered questions about AgNP transformation in environmental settings such as the rate and extent of the sulfidation, as well as the aggregation and dissolution behavior.

We found that the extent of sulfidation of faceted AgNPs has a strong preference on the crystallographic faceting. Passivation layers with nm-scale layer thicknesses developed on {111} surfaces, and Ag_2_S nucleation and growth proceeded inward from the vertices of the AgNPs along the 〈110〉 directions. Our extensive NBED results clearly demonstrated that the crystallinity of Ag was preserved, and that the precipitated Ag_2_S domains were also fully crystalline in all the TEM aliquots. TEM conclusively demonstrated that sulfidation at S/Ag = 0.72 is a slow process with a large fraction of silver in the middle of the AgNPs remaining unreacted after 24 h of sulfidation.

*In situ* SAXS and XRD allowed simultaneous determination of the real-time morphological changes of the AgNPs and the rate of sulfidation. Both SAXS and XRD results strongly indicate that sulfidation follows first-order reaction kinetics. The changes in particle size extracted from SAXS analysis and the conversion kinetics extracted from XRD analysis follow similar kinetic rates, establishing the coupling between particle morphology and extent of atomic structure transformation. The rates can be used to serve as benchmarks to validate thermodynamic models and potentially enable high-fidelity predictions of the fate and environmental impacts of AgNPs. Importantly, SAXS results also present definitive evidence proving at a high S/Ag ratio of 0.72, the lack of aggregation in the entire duration of the *in situ* study in this model system involving common ligands and natural organic matter.

We also probed the long-term fate of the AgNPs under different S/Ag ratios using *ex situ* SAXS/XRD. We found that the converted volume of Ag (Ag_2_S) is linearly related to the initial availability of sulfide in the range of S/Ag between 0 and 1 with the individual characteristic of the AgNPs well preserved, suggestive of sulfidation being a well-regulated reaction. A careful analysis also establishes that the silver mass in the AgNP and transformed Ag/Ag_2_S NP is preserved. This result strongly indicates no dissolved Ag^+^ ions were leached into the solution, a result with profound environmental implication.

While our results are specific to the materials system under investigation, we emphasize that the combined nondestructive methodology can be readily extended to directly probe and unfold the structure transformation pathway and the relevant kinetics in a broad range of model ENM systems. TEM allows in-depth characterization of localized structures in the ENMs, and *in situ* SAXS/XRD provides statistically significant knowledge regarding the kinetic rate and the extent of the transformation. Together, these complementary techniques present a detailed structure transformation landscape that is critically missing in our understanding of the behaviors of ENMs.^5,6,24^ It is also important to acknowledge that due to the contrast mechanism of both TEM and X-ray scattering, this methodology is sensitive to the transformation in the metallic core alone and cannot reveal deterministic information related to the surfactant (organic) and nanoparticle (inorganic) interface, which as an influential critical review puts, “(surface structure) is a major unknown factor because there are currently no methods available for determining nanoparticle surface structure at the molecular level”.^24^ Recent developments in attenuated total reflectance-Fourier transform infrared spectroscopy have shown promise in the quantitative determination of molecular adsorption on various ENMs.^28,72^ Use of the H/D isotope contrast effect in neutron scattering methods may also provide insights regarding the surfactant–surface interaction. Together with the structure evolution of the metallic core enabled by the methodology presented in this paper, we may be positioned to understand the contributing factors that determine the fate and elucidate the risks of ENMs in complex environmental settings.

## Conflicts of interest

There are no conflicts to declare.

## Supplementary Material

NA-001-C8NA00103K-s001

NA-001-C8NA00103K-s002

NA-001-C8NA00103K-s003
