# Supplementary material for: Transformation of engineered nanomaterials through the prism of silver sulfidation
Source: Nanoscale Adv. 2018 Aug 17;1(1):241–53. doi: 10.1039/c8na00103k (PMC6605090; doi:10.1039/c8na00103k)
Supplement: NA-001-C8NA00103K-s001 [file NA-001-C8NA00103K-s001.pdf]

Electronic Supplementary Material (ESI) for Nanoscale Advances.
This journal is © The Royal Society of Chemistry 2018

Supporting Information

Transformation of Engineered Nanomaterials through the Prism of Silver Sulfidation

Fan Zhang, Andrew J. Allen, Aaron C. Johnston-Peck, Jingyu Liu, and John M. Pettibone

Material Measurement Laboratory, National Institute of Standards and Technology
Gaithersburg, MD 20899, USA

S1: Details of USAXS Measurement

The USAXS instrument uses Bonse-Hart type crystal optics to extend the lower end of the scattering q range of SAXS to 10^{-4} Å^{-1}, a value that is normally inaccessible to SAXS instruments in the pinhole geometry. Here, $q = 4\pi/\lambda \times \sin(\theta)$, where λ is the X-ray wavelength and θ is one half of the scattering angle 2θ. The USAXS instrument measures absolute scattering intensity, i.e., the differential scattering cross section $d\Sigma/d\Omega$, where Σ is the scattering cross section per unit sample volume and Ω is the solid angle. For the experiments reported in this study, we conducted the USAXS experiments in the standard 1-D collimated geometry. X-ray wavelength λ was 0.05904 nm, which corresponds to an X-ray energy of 21.0 keV. The relative wavelength spread $\Delta\lambda/\lambda \approx 10^{-4}$. At the sample position, the X-ray photon flux was $\approx 10^{13}$ photon/s/mm^2. The beam size for USAXS experiment was 0.4 mm × 0.4 mm.

The SAXS and XRD experiments were conducted using two standalone Pilatus 2-D area detectors (Model: 100K-S, Dectris, Baden, Switzerland), also equipped at the beamline.[1] The beam size in both configurations was 0.2 mm × 0.2 mm to match the pixel size of the detectors (172 µm × 172 µm) and to provide the best instrumental resolution. The sample to detector distances were 533.4 mm and 244.1 mm for SAXS and XRD experiments, respectively. The q calibration was conducted using silver behenate and NIST Standard Reference Material, SRM 660 (LaB_6: lanthanum hexaboride)[2], respectively. The combination of USAXS, SAXS, and XRD measurements covers a continuous q range from $\approx 1 \times 10^{-4}$ Å^{-1} to ≈ 5 Å^{-1}, and an intensity range of over 10 decades. More details about this type of combined measurements can be found elsewhere. [3]

Synchrotron measurements in this study were of two kinds, *in situ* and *ex situ*. The duration of the *in situ* experiment (hours) is limited by the availability of synchrotron beam time. Therefore, the experimental conditions of the *in situ* component are optimized to understand the early stage of the AgNP sulfidation kinetics. Furthermore, we adopted an empirical criterion to determine the measurement duration – when the last three sets of USAXS and XRD data showed no significant difference, we assumed that the kinetics was slow enough to allow the termination of the *in situ* experiments. The *ex situ* measurements, on the other hand, were aimed at understanding the nature of the final product after a prolonged silver sulfidation process (up to several days).

The *in situ* experiment reported in this study was conducted using a custom-made flow cell system, which enabled continuous flow of the sample. The flow cell includes a 1.5 mm-diametered quartz capillary tube (wall thickness 10 µm) and an automatic pump that maintains steady flow rate over time. The usage of a flow cell, in comparison with a commonly used sealed reaction cell, has the advantage of significantly reducing the amount of the time that the sample interacts with the ionizing X-ray beam while ensuring

sample consistency and measurement integrity. More details about the flow cell diagram and setup can be found elsewhere. [4]

In the *in situ* study, we followed the procedure detailed below:

1. 1.5 mL of pH adjusted (pH7) FA solution (64.86 mg FA was dissolved in 0.7 mL water and 0.8 mL 10-folds concentrated MHRW) was added into 6 mL of purified AgNP suspension (2.16 mg mL^{-1}) in a vial to achieve a mass ratio of FA to AgNPs at 5:1. After vigorous shaking for 1 min. The combined USAXS/SAXS/XRD dataset was collected as the baseline of the initial (pristine) state of the AgNP suspension. The data acquisition times for USAXS, SAXS, and XRD were 90 s, 30 s, and 30 s, respectively. For consistency, this set of acquisition time was used throughout the X-ray study.
2. We dissolved 0.022 g crystalline $Na_2S{\cdot}9H_2O$ in 0.5 mL of DI water. The resulting molar ratio between S and Ag was approximately 0.72:1, i.e., a slight overabundance of S to ensure the availability of S^{2-} required for complete AgNP sulfidation.
3. We added the dissolved Na_2S solution to the AgNP suspension and started the *in situ* experiments. Data collection was achieved in a repeated sequence of USAXS, SAXS, and XRD measurements. Each set of USAXS/SAXS/XRD measurement took $\approx$ 5 min (for simplicity, we will refer to combined USAXS/SAXS as SAXS from this point on). For successive sets, the sample position was shifted downward by 0.4 mm along the quartz capillary while maintaining the beam centered at the middle of the capillary. This was to minimize any risk of material deposition on the capillary surface due to prolonged X-ray irradiation.

The *ex situ* samples were prepared approximately 10 days before the synchrotron measurements following a similar protocol to that of the *in situ* sample with the same starting materials. The main difference with the *ex situ* samples was that the molar ratio between S and Ag was adjusted systematically from 0 to 5. Details of these samples can be found in Table 1. The *ex situ* measurements were conducted using standard liquid cells available at the beamline. Necessary scattering data correction steps with liquid cells are described elsewhere.[5]

S2. Supporting TEM Data

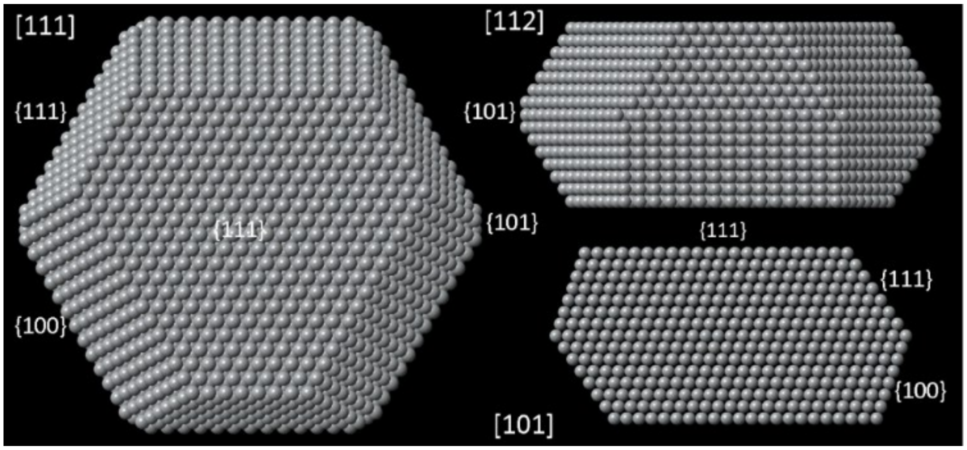

Figure S1. An atomistic model that illustrates the most commonly observed AgNP geometry. The surface is predominately terminated by {111} and {100} facets. We also found that the vertices of the particle

shape were nominally {110} terminated, and that the edges often had a rounded appearance resulting from surface steps and higher-order surface terminations, a feature identified earlier in a silver cube nanoparticle system.[6] Deviations from the geometry illustrated by this atomistic model were present and AgNPs with other types of common morphologies were observed, such as penta-twinned nanoparticles,[7] and polycrystalline particles without coherent grain boundaries (i.e., twins) with irregular morphologies[8-9]. While the shapes of these particles may differ from the model, their sizes were similar.

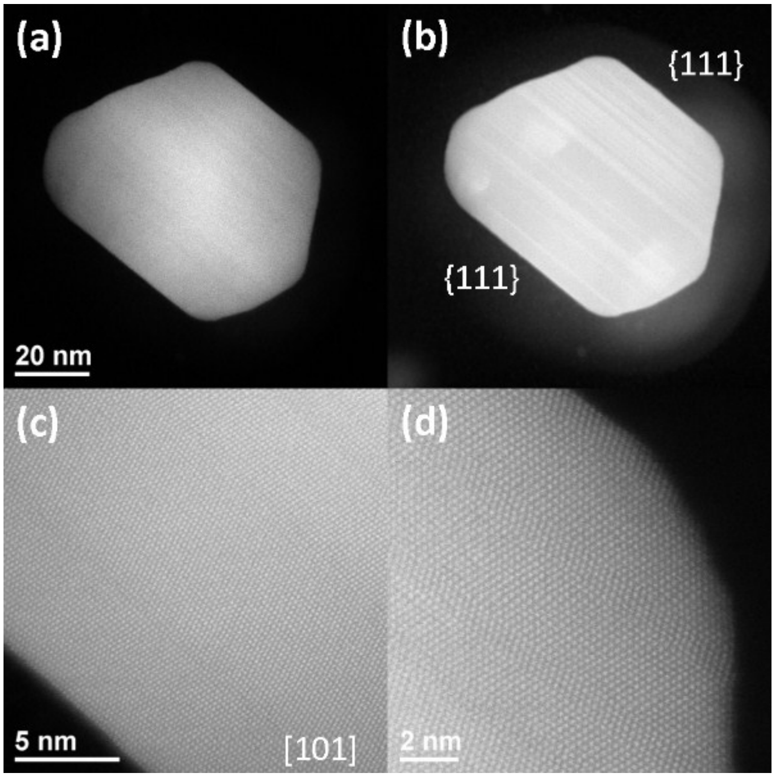

Figure S2. HAADF (a) and LAADF (b) images of a Ag particle. Striped contrast running parallel to the top and bottom {111} surfaces in the LAADF image is due to planar defects. High resolution image (c) identifies these planar defects as twins. Near edges the rounding, as seen in (a) and (b), is in part due to higher order surface terminations (d).

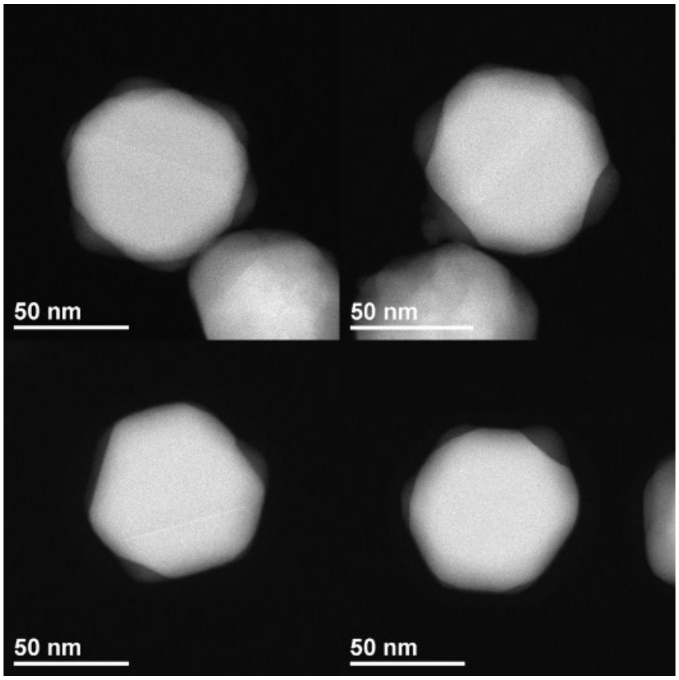

Figure S3. Additional examples of Ag_2S/Ag nanoparticles after 8 min of reaction. The particles are oriented to the [111] zone axis.

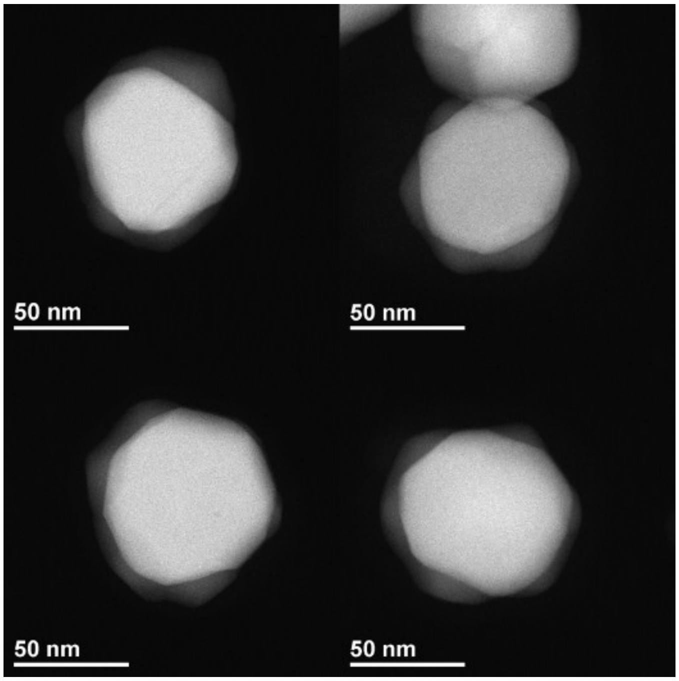

Figure S4. Examples of Ag_2S/Ag nanoparticles after 30 min of reaction. The particles are oriented to the [111] zone axis.

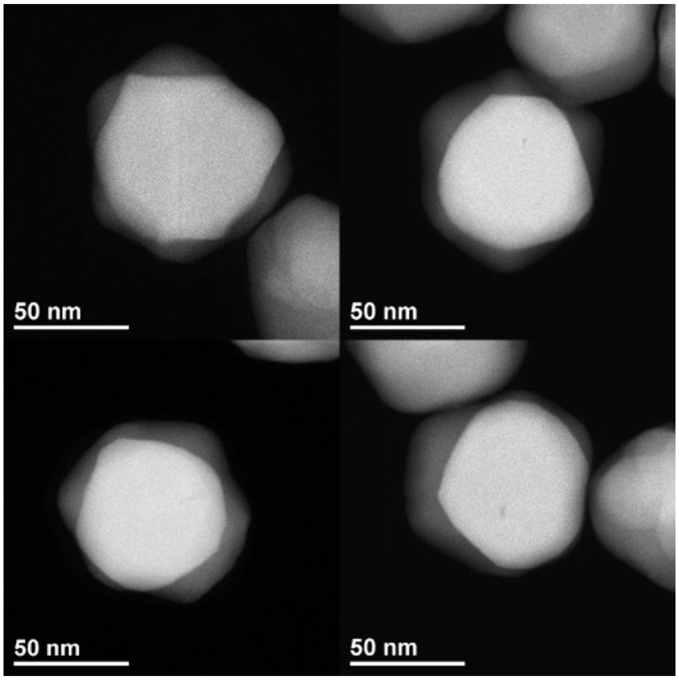

Figure S5. Additional examples of Ag_2S/Ag nanoparticles after 1 h of reaction. The particles are oriented to the [111] zone axis.

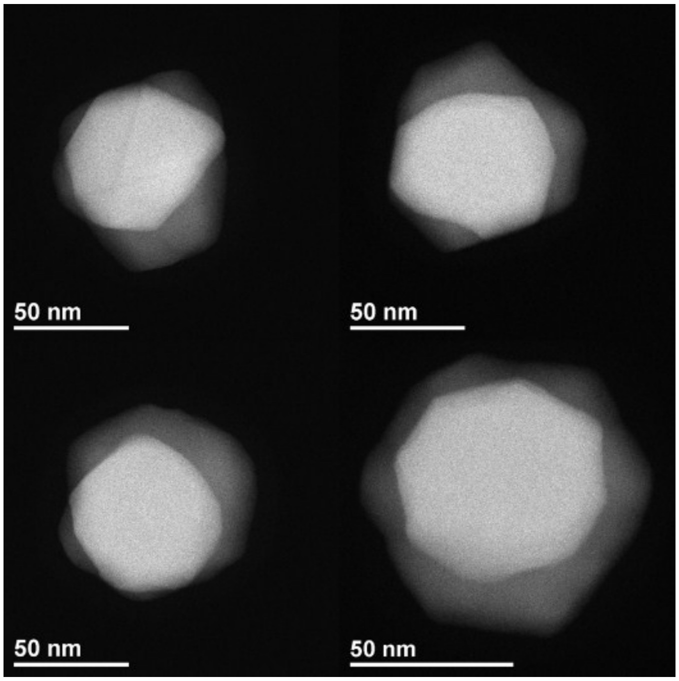

Figure S6. Additional examples of Ag_2S/Ag nanoparticles after 8 h of reaction. The particles are oriented to the [111] zone axis.

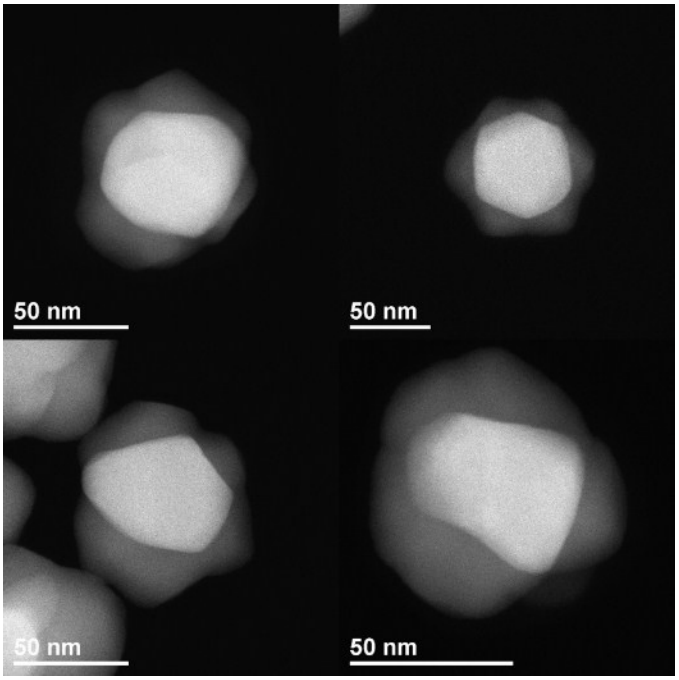

Figure S7. Additional examples of Ag_2S/Ag nanoparticles after 24 h of reaction. The particles are oriented to the [111] zone axis.

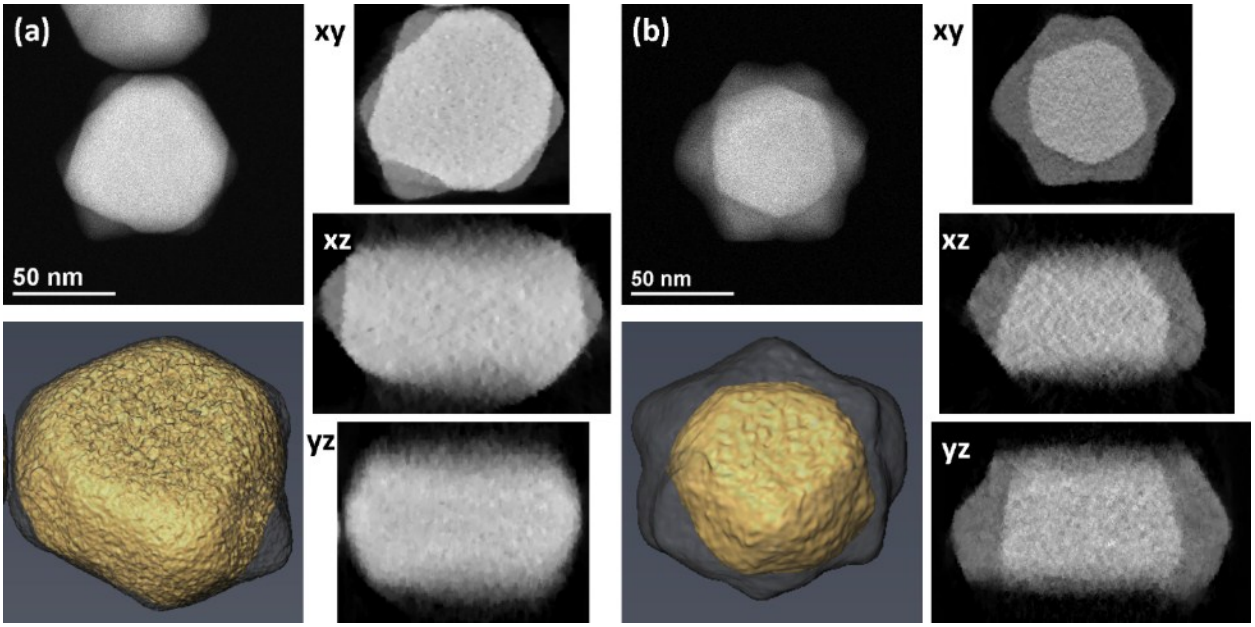

Figure S8. HAADF image and tomographic reconstruction of two Ag_2S/Ag particles after 8 min (a) and 24 h (b). Three orthogonal slices from the tomogram are shown along with an image showing two isosurfaces: the transparent gray surface represents the particle surface, while the opaque yellow surface is the interface between the Ag and Ag_2S. After 8 min Ag_2S has nucleated at the particle vertices. After 24 h the Ag_2S regions have grown and impinged on one another but a Ag core remains.

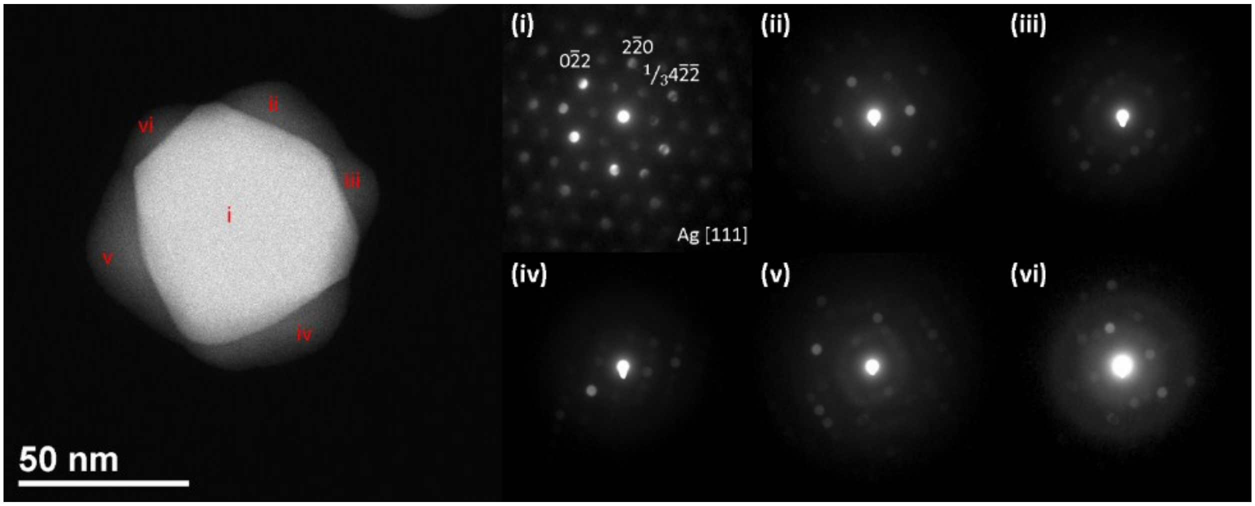

Figure S9. NBED patterns from the Ag and individual Ag_2S domains of a Ag_2S/Ag particle. No consistent crystallography orientation relationship relating the Ag and Ag_2S was identified. A nonlinear histogram adjustment was applied to the NBED patterns to highlight the presence of weak features.

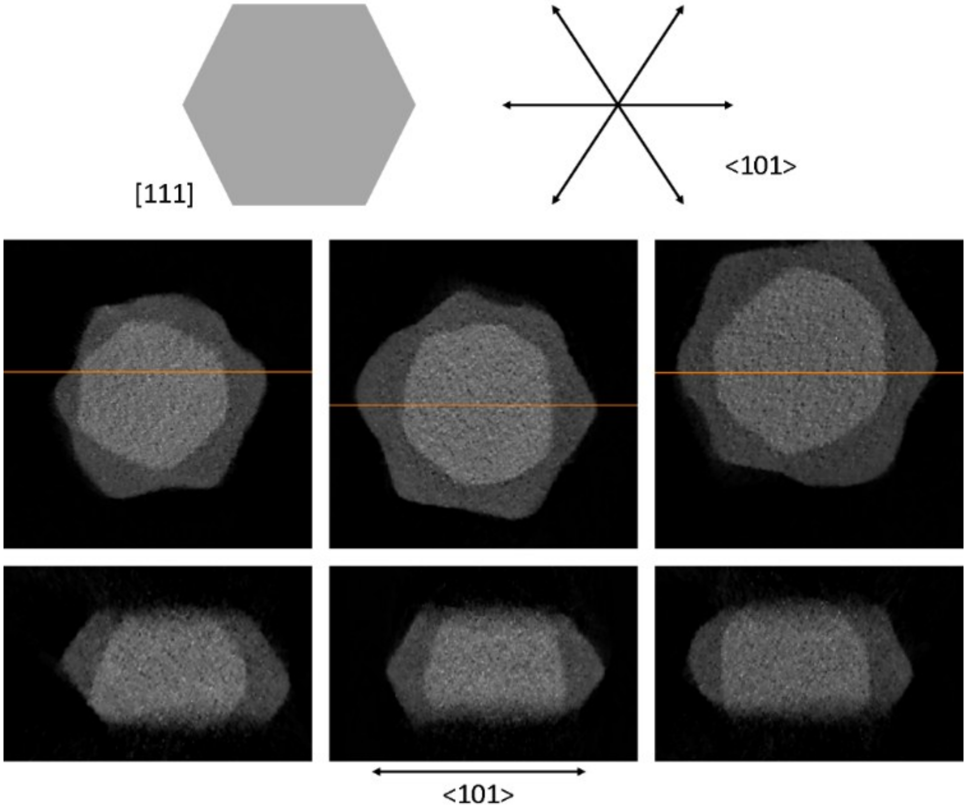

Figure S10. A model of a Ag nanoparticle indicating the <101> crystallographic directions. Slices from a tomographic reconstruction from a 24 h Ag_2S/Ag particle depict the interface between the Ag and Ag_2S. The top images with the orange line indicates the slice position of the bottom image. The bottom images are approximately parallel to the <101> directions. The interface between the Ag and Ag_2S is not always orthogonal to the <101> directions indicating that the reaction front proceeds on multiple planes.

S3: USAXS Analysis

X-ray scattering is a class of nondestructive structural characterization technique that has generated broad impact in a diverse range of research fields, due to its unique capability to characterize the materials structure and morphology in real time and in realistic sample environments. Synchrotron-based SAXS and XRD, in particular, due to its high flux density, tunable X-ray energy, broad applicable size range, has been increasingly recognized as an important analytical tool in the field of nanoparticle research to investigate kinetics associated with particle synthesis, transformation, aggregation, and agglomeration.[10] Despite these advantages, combined SAXS/XRD studies of the kinetics of the environmental transformations of ENMs are very limited. In our *in situ* SAXS/XRD study, we aimed to use SAXS to investigate the morphological transformation kinetics of the AgNPs, and use XRD to investigate the structural transformation of the AgNPs, with the hope that complimentary information across a sub-nanometer to micrometer length scale could be acquired in serving to elucidate the rate and extent of silver sulfidation.

To determine the starting size distribution of the AgNPs prior to sulfide addition, pristine AgNPs suspensions were examined. The 1D collimated, absolutely calibrated SAXS data is shown in Figure 7. We performed the SAXS data reduction and analysis using the standard SAXS analysis packages *Indra* and *Irena,*[11] developed at the APS. It is known that desmearing 1D collimated data is associated with numerical uncertainties.[5] Because of this, we performed data analysis on the slit-smeared data by convolving the modeled intensity $I(q)$ with the known instrumental slit-smearing prior to performing a least-squares regression analysis to the data. The slit size in the reciprocal space is determined by the X-ray wavelength, width of crystal rocking curves, sample to detector distance, and the horizontal dimension of the detecting area of the detector.

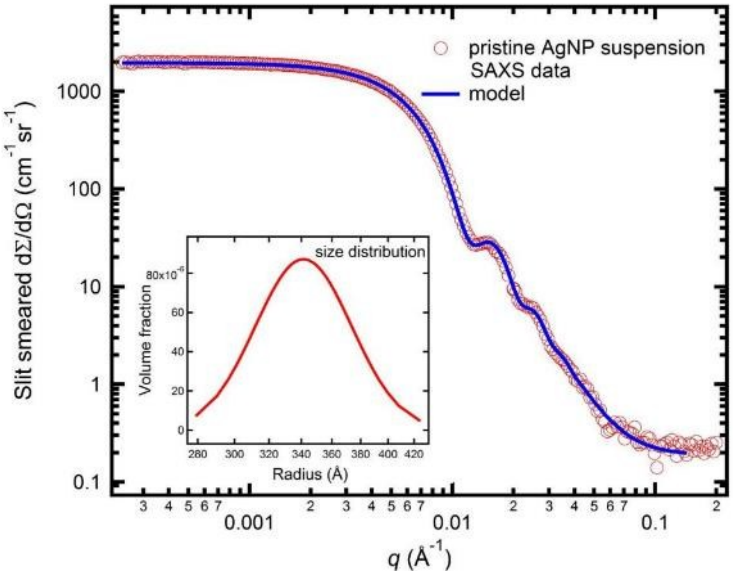

Figure S11: Slit-smeared SAXS profile of the pristine AgNP suspension. The measurement uncertainties in this figure, and henceforth, are smaller than the symbol sizes. The solid line shows the result from a

least-squares regression analysis using a model described in the text. The inset shows the starting volume size distribution (in radius) of the AgNPs.

The scattering curve in the inset of Figure S11 shows several Bessel oscillations, a characteristic that strongly indicates that the AgNPs, despite their shape irregularities observed under TEM, can be safely approximated as spherical particles for the purpose of small angle scattering analysis. This is not unexpected, given that the degree of asymmetry of the nanoscale AgNPs was low to start with. Furthermore, the AgNPs underwent rapid rotation in solution which creates a 3D gyrating profile resembling spheres. The Bessel oscillations also indicate that the size distribution of the AgNPs is narrow. In addition, we did not observe any upturn at the very low-q end of the SAXS profile, which shows that the unreacted AgNPs were well dispersed, a conclusion in good agreement with TEM results.

To quantify the size distribution of the AgNPs, we analyzed the scattering profile using a spherical scattering form factor, given by Eqn. (2) below:

$$F(q) == V(\rho - \rho_s) \times \left[\frac{3(\sin(qR) - qR\cos(qR))}{(qR)^3} \right], \quad (2)$$

where V is the volume of the sphere, R is the radius, ρ and ρ_s are the scattering length densities of the AgNPs and the solvent, respectively. The mass densities and scattering length densities of Ag and Ag_2S are listed in Table S1.

Table S1: Scattering length densities of Ag and Ag_2S

	Mass density (g/cm^3)	Scattering length density (10^{10} cm^{-2})
Ag	10.50	77.64
Ag_2S	7.23	54.46

We further assumed that the size of AgNPs follows a Gaussian volume size distribution, defined as

$$\phi(r) = \phi_0 \frac{1}{\sqrt{2\pi\sigma}} \exp\left[-\frac{(r - r_0)^2}{2\sigma^2} \right], \quad (3)$$

where r_0 is the mean radius, and σ is the Gaussian width. The volume fraction of the AgNPs in the pristine solution is approximately 10^{-4}, which suggests that we can reasonably assume that interparticle interferences are negligible in the SAXS analysis and the observed intensity is simply the weighted sum of the scattering intensity of particles of a given size distribution. Mathematically speaking, the scattering intensity is written as

$$I_0(q) = \int \left[\Delta\rho \frac{4}{3}\pi r^3 \left[\frac{3(\sin(qr) - qr\cos(qr))}{(qr)^3} \right] \right]^2 \times \frac{1}{\sigma\sqrt{2\pi}} \exp\left[-\frac{(r - r_0)^2}{2\sigma^2} \right] dr \cdot \quad (4)$$

Results from a least-squares fitting using Eqn. (4) are shown in Figure S11 as a solid line. It is evident that the model fits the data well, which validates the choice of a spherical form factor and a Gaussian size

distribution. The inset of Figure S11 shows the volume size distribution of the AgNPs. We found that the mean radius is 34.3 nm, and the Gaussian width is 3.2 nm, confirming that the AgNPs had a very narrow size distribution.

From Eqn. (2), it can be inferred that that the first minimum in the scattering curves shown in Figures S11 and the inset of Figure 4 is directly related to the mean size of the AgNPs. Using this as a visual marker, we found that as time increases, the first minimum shifts to lower *q* at a rate that gradually decreases with overall sulfidation time, indicating a slowing down of the sulfidation transformation.

We performed quantitative analysis of the SAXS data in order to unveil the kinetic rate and the extent of transformation, with the latter playing a critical role in determining the bio-availability of silver in an environmental setting. Our TEM results show that the AgNPs have irregular shapes and that the sulfidation occurs at different crystallographic facets at different rates, which make it difficult to construct an exact scattering model. Instead, following the parsimony principle, we adopted an approach similar to the one used in a study of solution-mediated oxidation of iron nanoparticles,[12] where the mean size of the nanoparticles was analyzed assuming that they were spherical nanoparticles with homogeneous scattering contrast. Using this model, we were able to fit all the *in situ* SAXS datasets. A few examples of the detailed fitting are given in Figure S12-S14.

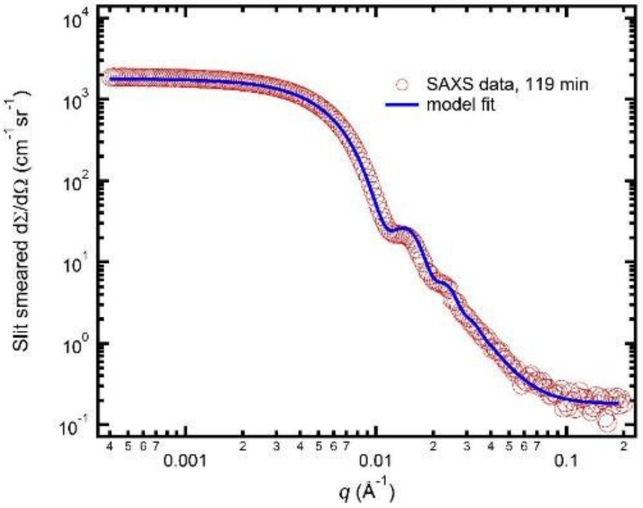

Figure S12: Slit-smeared SAXS profile of the AgNP suspension acquired at 119 min after sulfidation was initiated. The solid line shows the result from a least-squares regression analysis using a model described in the text.

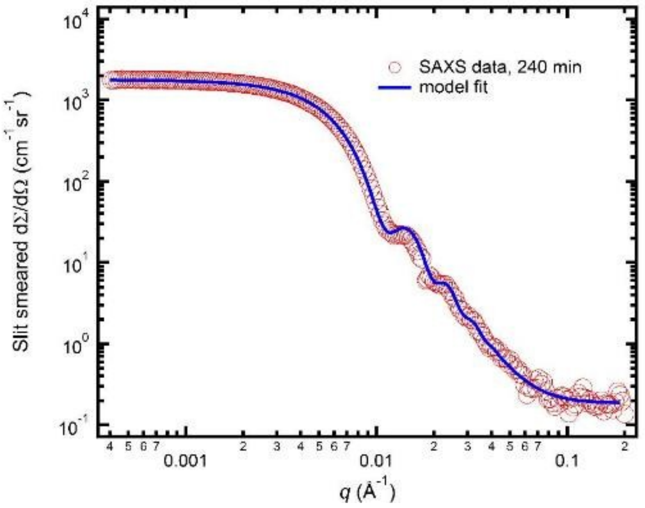

Figure S13: Slit-smeared SAXS profile of the AgNP suspension acquired at 240 min after sulfidation was initiated. The solid line shows the result from a least-squares regression analysis using a model described in the text.

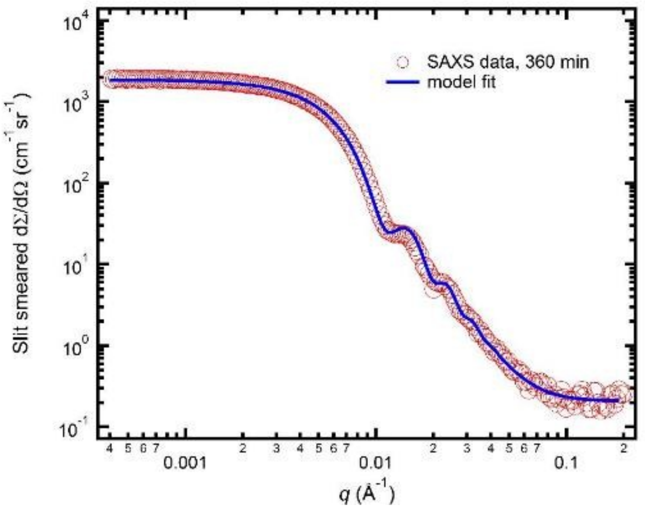

Figure S14: Slit-smeared SAXS profile of the AgNP suspension acquired at 360 min after sulfidation was initiated. The solid line shows the result from a least-squares regression analysis using a model described in the text.

Based on the SAXS analysis, we acquire time-dependent mean radius of the Ag/Ag_2S nanoparticles, as shown below:

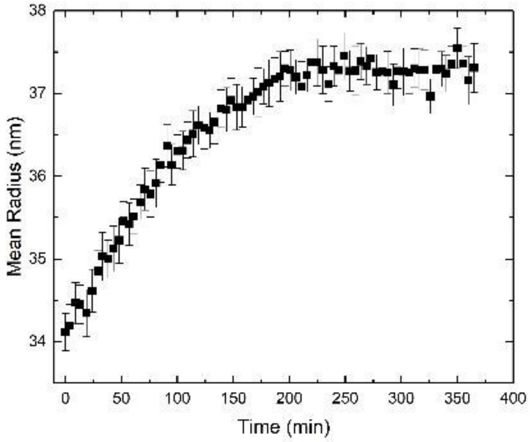

Figure 15: Time-dependent evolution of the mean radius of AgNPs upon sulfidation.

The mass density of Ag is ρ_{Ag} = 10.5 g/cm^3. The mass density of Ag_2S is ρ_{Ag2S} = 7.23 g/cm^3. The molar mass of Ag is m_{Ag} = 107.8 u, and S is m_S = 32.1 u. Based on the mass preservation conclusion drawn from the *ex situ* component of this work, we have, at any time t, the silver mass within a Ag/Ag_2S nanoparticle equals to that of a pristine AgNP.

At time t, assuming (1-x) amount of Ag is transformed to Ag_2S, the volume of Ag_2S in a Ag/Ag_2S NP is

$$V_{Ag2S} = (1\text{-}x)V_0\rho_{Ag}/\rho_{Ag2S}\times (m_S + 2m_{Ag})/ 2m_{Ag}. \quad (5)$$

Here, $V_0 = 4/3\pi r_0^3$. r_0 is the mean radius of the pristine AgNP.

Therefore, the total volume of the nanoparticle at time *t* is

$$V = xV_0 + V_{Ag2S} = 4/3\pi r_t^3. \quad (6)$$

Here, r_t is the mean radius of the Ag/Ag_2S nanoparticle at time *t*. Leading in the numerical values of the mass densities and molar masses, we have

$$x = (1.669V_0 – V)/0.669V_0. \quad (7)$$

Hence, from the mean radius of the nanoparticle, we were able to calculate the fraction of Ag inside an AgNP that is transformed to Ag_2S, as shown by Figure 4 in the main text.

S4: XRD data reduction

We note that due to the *in situ* nature of the experiment and the low concentration of the AgNPs in solution (compared to other scattering studies), despite the usage of very low-noise photon-counting detectors, the diffraction signal from the Ag_2S nanodomains remained weak. Traditional data reduction involved subtracting background from the diffraction data, which becomes challenging when weak data are on top of strong diffuse scattering signal from the solution and the sample cell, evident from the time-dependent evolution of the unnormalized XRD data shown in Figure S16. In order to highlight the

evolution of the weak Ag_2S diffraction peaks, we adopted an unusual approach of normalizing the diffraction data to the reference blank data, instead of the conventional subtraction. Mathematically, because a fixed reference blank dataset was consistently used, for a given peak, the integrated intensity evolution is preserved.

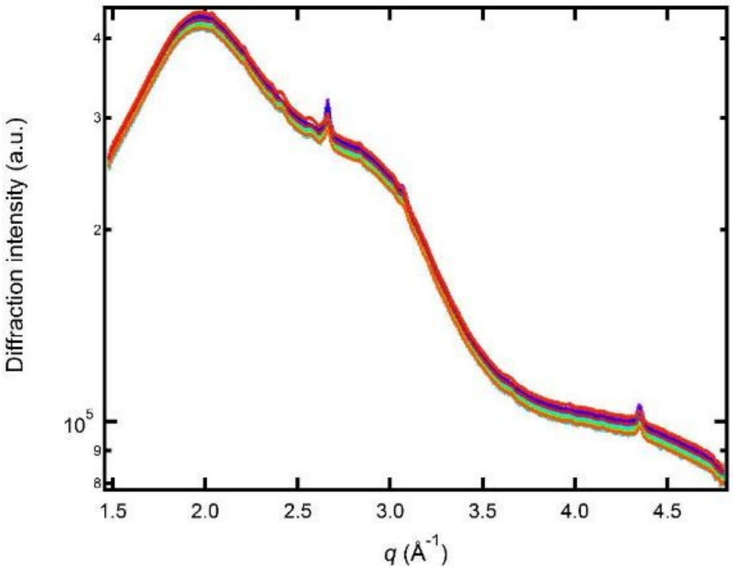

Figure S16: Time-dependent evolution of the unnormalized XRD patterns.

Examples of the unnormalized data and the normalization data are shown in S17 and S18, respectively.

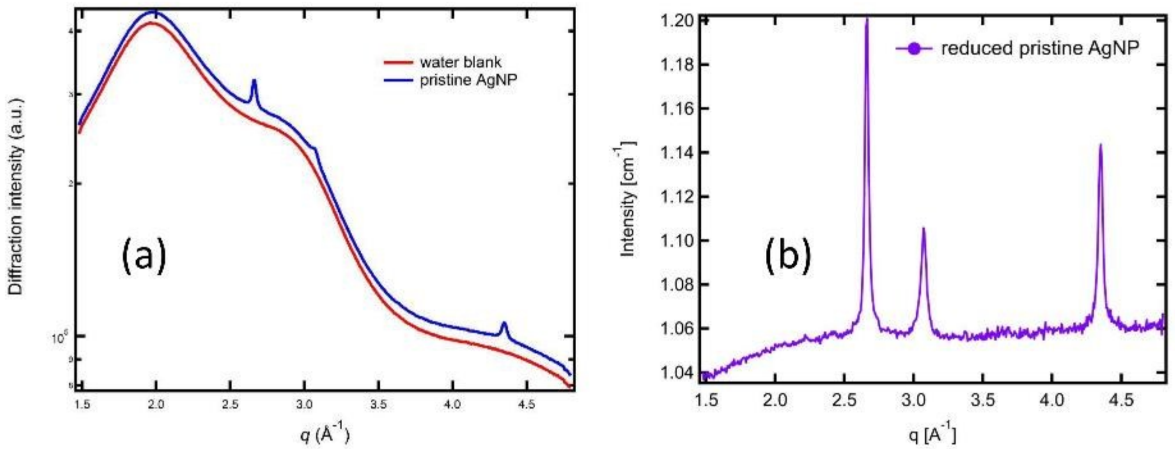

Figure S17: (a) A comparison between unnormalized XRD data acquired for the water blank and that for the pristine AgNP suspension. The diffraction signal from the AgNPs is weak and is on top of a complex scattering profile originating from the water blank. (b) shows the reduced XRD data, where sharp XRD

peaks characteristic of Ag lattice are clearly seen.

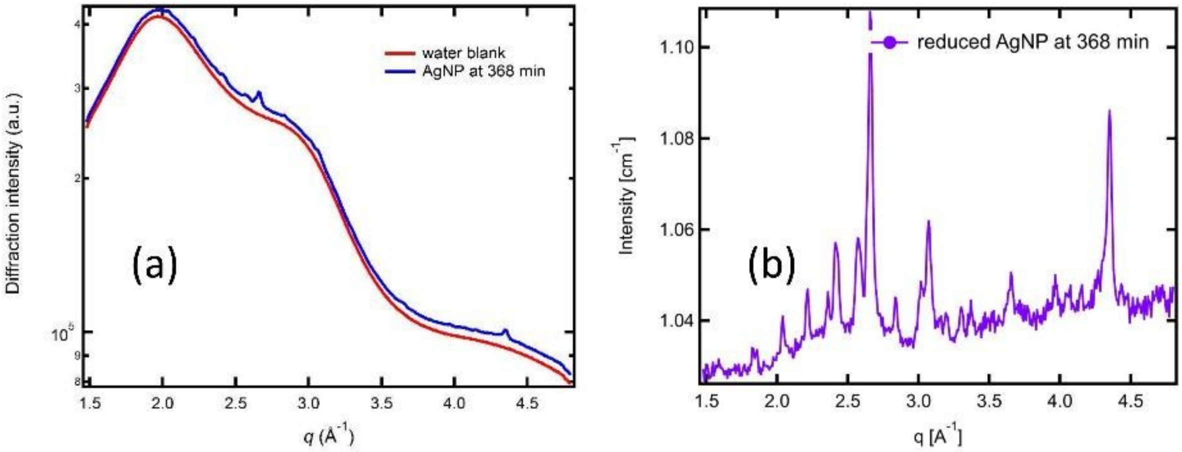

Figure S18: (a) A comparison between the unnormalized XRD data acquired for the water blank and that for the AgNP suspension acquired at 368 min after sulfidation was initiated. The Ag peaks from Figure S17 remain, and some very weak diffraction peaks develop on top of the complex scattering profile originating from the water blank. (b) shows the reduced XRD data, where sharp XRD peaks characteristic of Ag lattice and Ag_2S are clearly visible.

References:

1. Ilavsky, J.; Zhang, F.; Allen, A.; Levine, L.; Jemian, P.; Long, G., Ultra-Small-Angle X-Ray Scattering Instrument at the Advanced Photon Source: History, Recent Development, and Current Status. *Metallurgical and Materials Transactions A* **2013,** *44*, 68-76.
2. Black, D. R.; Windover, D.; Henins, A.; Filliben, J.; Cline, J. P., Certification of Standard Reference Material 660b. *Powder Diffraction* **2011,** *26*, 155-158.
3. Zhang, F.; Levine, L. E.; Allen, A. J.; Campbell, C. E.; Creuziger, A. A.; Kazantseva, N.; Ilavsky, J., In Situ Structural Characterization of Ageing Kinetics in Aluminum Alloy 2024 across Angstrom-to-Micrometer Length Scales. *Acta Materialia* **2016,** *111*, 385-398.
4. Allen, A. J.; Hackley, V. A.; Jemian, P. R.; Ilavsky, J.; Raitano, J. M.; Chan, S.-W., In Situ Ultra-Small-Angle X-Ray Scattering Study of the Solution-Mediated Formation and Growth of Nanocrystalline Ceria. *Journal of Applied Crystallography* **2008,** *41*, 918-929.
5. Zhang, F.; Allen, A. J.; Levine, L. E.; Tsai, D.-H.; Ilavsky, J., Structure and Dynamics of Bimodal Colloidal Dispersions in a Low-Molecular-Weight Polymer Solution. *Langmuir* **2017,** *33*, 2817-2828.
6. Xia, X.; Zeng, J.; Oetjen, L. K.; Li, Q.; Xia, Y., Quantitative Analysis of the Role Played by Poly (Vinylpyrrolidone) in Seed-Mediated Growth of Ag Nanocrystals. *Journal of the American Chemical Society* **2012,** *134*, 1793-1801.

7. Murshid, N.; Kitaev, V., Role of Poly (Vinylpyrrolidone)(Pvp) and Other Sterically Protecting Polymers in Selective Stabilization of {111} and {100} Facets in Pentagonally Twinned Silver Nanoparticles. *Chemical communications* **2014,** *50*, 1247-1249.
8. Pal, A.; Shah, S.; Devi, S., Microwave-Assisted Synthesis of Silver Nanoparticles Using Ethanol as a Reducing Agent. *Materials Chemistry and Physics* **2009,** *114*, 530-532.
9. Kaegi, R.; Voegelin, A.; Ort, C.; Sinnet, B.; Thalmann, B.; Krismer, J.; Hagendorfer, H.; Elumelu, M.; Mueller, E., Fate and Transformation of Silver Nanoparticles in Urban Wastewater Systems. *Water research* **2013,** *47*, 3866-3877.
10. Li, T.; Senesi, A. J.; Lee, B., Small Angle X-Ray Scattering for Nanoparticle Research. *Chemical reviews* **2016,** *116*, 11128-11180.
11. Ilavsky, J.; Jemian, P. R., Irena: Tool Suite for Modeling and Analysis of Small-Angle Scattering. *Journal of Applied Crystallography* **2009,** *42*, 347-353.
12. Sun, Y.; Zuo, X.; Sankaranarayanan, S. K.; Peng, S.; Narayanan, B.; Kamath, G., Quantitative 3d Evolution of Colloidal Nanoparticle Oxidation in Solution. *Science* **2017,** *356*, 303-307.
